# Effects of physical activity intervention on active ageing and physical function among older adults: a systematic review and meta-analysis

**DOI:** 10.3389/fpsyg.2026.1958928

**Published:** 2026-10-12

**Authors:** Wenxuan Wang, Liangcai Yao

**Affiliations:** School of Physical Education, Qiqihar University, Qiqihar, Heilongjiang, China

**Keywords:** active aging, older adults, physical activity intervention, physical exercise, physical function

## Abstract

**Background:**

Although the health benefits of physical activity for older adults have attracted extensive scholarly attention, existing systematic reviews mostly focus on a single outcome domain. Few studies have simultaneously performed quantitative synthesis of Active Ageing and physical function within a unified analytical framework.

**Objective:**

Registered on (PROSPERO registration: CRD420261465400), this systematic review and meta-analysis comprehensively evaluated the effects of physical activity intervention on Active Ageing (global Active Ageing, mental health, social participation, functional health) and physical function (SPPB, chair stand test, TUG, gait speed, handgrip strength, balance) among older adults.

**Methods:**

Three databases including PubMed, Web of Science and CNKI were searched to retrieve peer-reviewed Chinese and English randomized controlled trials (RCTs) published up to July 1, 2026. The Cochrane Risk of Bias tool was adopted to assess the methodological quality of included studies.

**Results:**

A total of 25 RCTs with a combined sample size of 7,861 participants were incorporated. For the Active Ageing domain, global Active Ageing (SMD = 1.38, 95%CI = [0.54, 2.22], *p* = 0.001), social participation (SMD = 1.03, 95%CI = [0.87, 1.20], *p* < 0.00001) and mental health (SMD = 0.6, 95%CI = [0.17, 1.03], *p* = 0.006) exhibited statistically significant improvements, whereas functional health showed no significant between-group difference (*p* = 0.07). In terms of physical function, significant improvements were observed in SPPB (SMD = 0.33, 95%CI = [0.07, 0.58], *p* = 0.01), chair stand test (SMD = 0.37, 95%CI = [0.10, 0.63], *p* = 0.006) and TUG (SMD = -0.68, 95%CI = [−1.16, −0.20], *p* = 0.006). No significant effects were detected for gait speed, handgrip strength and balance. Subgroup analyses provided exploratory insights into potential moderators of intervention effects. For mental health outcomes, twice-weekly interventions yielded significant within-group improvements, whereas three-times-weekly interventions did not reach statistical significance. However, formal between-subgroup comparison showed no statistically significant difference in effect magnitude across frequency strata, so these frequency-based findings remain hypothesis-generating and require confirmation in larger studies.

**Conclusion:**

Physical activity intervention significantly improves multiple dimensions of Active Ageing and partial physical function indicators in older adults, yet the ameliorative effects present domain-specific and indicator-specific characteristics. These findings yield preliminary evidence for physical-activity-related benefits across select active-aging and physical-function domains, though optimal intervention modality, frequency, intensity and duration remain undefined, precluding definitive program-design guidance from current evidence.

## 1 Introduction

The global population is undergoing population aging at an unprecedented rate. The global population is aging at an unprecedented rate. Globally, the proportion of adults aged 60 years and older is projected to rise from 12% in 2000 to 22% by 2050, with the number of people aged 80 years and older expected to increase almost fivefold over the same period (Parant, 1990). This demographic shift places substantial pressure on public health and social security systems worldwide. This demographic structural shift has exerted profound impacts on global public health and social security systems. Against this backdrop, Active Ageing is defined as a process of optimizing opportunities for health, participation and security to enhance quality of life (Haighton et al., 2018). Rather than a unidimensional construct, Active Ageing features a multidimensional framework that generally encompasses mental health, social participation, functional health and global Active Ageing. As a vital component of health status, physical function is correlated with elevated risks of falls, fractures, subsequent mobility limitation and hospitalization among middle-aged and Older Adults (Lin et al., 2023). Key indicators of physical function mainly include the SPPB, balance, handgrip strength, gait speed and TUG. According to the WHO, healthy aging largely relies on the preservation of intrinsic capacity, which refers to the aggregate of all physical and mental capacities of an individual (Parant, 1990). Accordingly, physical function constitutes a core determinant of healthy aging for older adults. Physical activity intervention not only improves physical and mental well-being among older adults (Lai et al., 2022; Macdonald et al., 2024), but also maintains a close correlation with physical function. Multiple empirical studies have confirmed that higher levels of physical activity, motor competence and physical fitness are positively correlated with superior physical function (Santanasto et al., 2017; Yoon et al., 2018). Nevertheless, the specific patterns of such correlation across the two domains of Active Ageing and physical function remain controversial in existing scholarship.

In recent years, a number of systematic reviews have performed quantitative syntheses of the health effects of physical activity on single or a small number of outcome domains among older adults. For example, several meta-analyses have examined the impacts of physical activity on physical function indicators (e.g., gait speed, balance, SPPB) in older adults and verified moderate intervention improvements (de Labra et al., 2015; Sherrington et al., 2020). Other reviews have concentrated on the beneficial effects of physical activity on mental health or cognitive function of older adults (Ge et al., 2026). Although these studies imply potential multi-domain health benefits, they fail to conduct comprehensive quantitative pooling due to limitations in the quantity and methodological quality of eligible trials. Combined with the above scholarly controversies, obvious gaps remain in existing relevant systematic reviews. First, few reviews take Active Ageing (covering mental health, social participation, functional health and global Active Ageing) as the core outcome framework to simultaneously and separately synthesize effect sizes across its four dimensions. Second, current reviews lack sufficient analyses of the moderating effects of intervention characteristics, including intervention type, weekly frequency and session duration. Distinct forms of physical activity [aerobic exercise (Antunes et al., 2005), resistance training (Pedersen et al., 2017) and general physical activity (Figueira et al., 2012)], may generate heterogeneous effects across different outcome domains. Moreover, identical intervention frequencies [twice weekly (Figueira et al., 2012) versus three times weekly (Sadjapong et al., 2020)] do not necessarily yield consistent outcomes for Active Ageing and physical function. Third, high-quality randomized controlled trials (RCTs) focusing on physical activity intervention for older adults published within the past 3 years (Song et al., 2026; Yang et al., 2025), have not been incorporated into previous review analyses, which substantially weakens the causal interpretability of prior findings. Accordingly, this systematic review and meta-analysis aims to integrate data from randomized controlled trials published up to July 1, 2026. It simultaneously evaluates the effects of physical activity intervention on four dimensions of Active Ageing (global Active Ageing, mental health, social participation, functional health) and six indicators of physical function (SPPB, chair stand test, TUG, gait speed, handgrip strength, balance) among older adults aged ≥60 years. Where data availability permits, subgroup analyses will be conducted to explore the potential moderating roles of intervention type and weekly frequency for partial outcomes. In addition, inconsistencies across different RCTs in sample size, intervention protocols and measurement tools further hinder the formation of unified research conclusions.

To address the aforementioned scholarly disputes and research gaps, the present study resolves two core theoretical debates in this field from a theoretical perspective. First, it responds to the controversy over whether the facilitating effects of physical activity on Active Ageing are universal or domain-specific. Second, it clarifies whether intervention effects remain consistent or differ under various weekly intervention frequencies. This study therefore hypothesizes that physical activity intervention acts not only as a favorable contributor to Active Ageing but also exerts ameliorative impacts on physical function among older adults. From a practical perspective, this research provides clear guidance for designing physical activity intervention programs to promote Active Ageing and maintain physical function in older populations. On the basis of existing empirical evidence, this study further expects to confirm that physical activity intervention markedly elevates the overall level of Active Ageing (Chi et al., 2021) and produces significant improvements in multiple physical function indicators (Yoon et al., 2018). Integrating both Active Ageing and physical function into a unified analytical framework enables a comprehensive understanding of the multi-domain pathways through which physical activity shapes the health of older adults. By exclusively enrolling RCTs, this meta-analysis synthesizes evidence from multiple trials to improve the generalizability of findings compared with single RCT studies. Relative to meta-analyses incorporating non-randomized studies, the exclusive inclusion of RCTs upgrades the grade of evidence and strengthens causal interpretability.

The core objective of this study is to focus on older populations and clarify the effects of physical activity intervention on their Active Ageing (including mental health, social participation, functional health and global Active Ageing) and physical function (including SPPB, chair stand test, TUG, gait speed, handgrip strength and balance). Meta-analytic synthesis is adopted to deeply explore the underlying mechanisms through which physical activity intervention improves physical and mental well-being among older adults. After identifying the direction of intervention effects, this study further quantifies the promoting magnitude of physical activity intervention on Active Ageing and its ameliorative effects on physical function.

## 2 Methods

### 2.1 Literature search strategy

This systematic review and meta-analysis was reported in strict accordance with the 2020 Preferred Reporting Items for Systematic Reviews and Meta-Analyses (PRISMA) guideline. The primary objective of literature searching was to comprehensively and systematically retrieve eligible randomized controlled trials (RCTs) relevant to the research topic, ensuring comprehensiveness, systematicness and reproducibility of the search strategy and preventing omission of eligible studies. Database selection was guided by the core disciplinary scope of this review (gerontology, exercise science, and public health) and aligned with prevailing methodological standards for systematic reviews in physical activity and aging research. Three complementary core databases were selected to maximize coverage of the global evidence base while ensuring linguistic and disciplinary balance: (1) PubMed: The leading biomedical database indexing the full spectrum of geriatric health, rehabilitation, and clinical exercise science research, with rigorous coverage of randomized controlled trials (RCTs) in human health; it serves as the primary source for English-language clinical evidence in this review. (2) Web of Science Core Collection: A comprehensive multidisciplinary database covering peer-reviewed literature across the natural sciences, social sciences, and humanities, which captures international RCTs in sport science, social gerontology, and public health that may not be fully indexed in PubMed. (3) China National Knowledge Infrastructure (CNKI): The most authoritative and comprehensive database for Chinese-language academic research, which ensures adequate representation of RCTs conducted in Chinese community settings, a population group underrepresented in many Western-focused systematic reviews. This tripartite database strategy has been widely adopted in prior systematic reviews examining physical activity interventions in older adults (Lin et al., 2023; Sherrington et al., 2020), and *post hoc* verification confirmed that 23 out of the 25 finally included studies (92%) were indexed in at least two of the three databases, indicating strong coverage of the core eligible evidence base. To complement electronic database searching and minimize retrieval bias, we also manually screened the reference lists of all included studies and relevant systematic reviews to identify potentially eligible trials that may have been missed by database searches. The search period spanned from the inception of each database to July 1, 2026. Our electronic database searches captured all records indexed in PubMed, Web of Science, and CNKI up to July 1, 2026. Excluded records during title-abstract and full-text screening were removed based on our predefined PICO-based eligibility criteria, rather than because of search-date restrictions. Full raw search output files from each database are provided within the Supplementary materials of this manuscript. A combined strategy of controlled vocabulary (subject headings) and free-text synonyms was adopted to develop database-specific search strings covering two core conceptual dimensions: intervention (physical activity intervention) and primary outcomes (active aging, physical function). This approach balances sensitivity and specificity, minimizing retrieval bias while maximizing capture of eligible studies. For each database, search terms were adapted to the platform’s specific indexing syntax. PubMed searches incorporated Medical Subject Headings (MeSH) with explosion to capture hierarchical synonyms, Web of Science searches used topic field matching with wildcard operators, and CNKI searches applied Chinese subject term indexing with synonym expansion. Boolean operators were applied consistently across all databases, with synonyms within the same dimension connected by “OR” and terms from different dimensions combined by “AND.” Standardized filters were applied to restrict results to human research, peer-reviewed journal articles, and publications from database inception to July 1, 2026, alongside additional age and language filters matched to the study eligibility criteria. To complement electronic database searches and minimize retrieval bias, we also manually screened the reference lists of all included studies and 12 relevant systematic reviews identified during screening to identify potentially eligible trials that may have been missed by database searches. Complete database-specific search strings, including all controlled vocabulary terms, free-text synonyms, Boolean logic, and filter settings, are provided in full in the Supplementary materials of this manuscript.

Notably, five discipline-specific databases were not included in the electronic search: Scopus, Cochrane Library, SPORTDiscus, CINAHL, and PsycINFO. The rationale for this prioritization is threefold. First, Scopus largely overlaps with Web of Science in journal coverage for exercise science and gerontology, with approximately 85–90% of core journals in this field indexed in both databases; adding Scopus would therefore yield predominantly duplicate records rather than unique eligible trials. Second, the Cochrane Library primarily hosts systematic reviews and Cochrane clinical trials, and the vast majority of RCTs indexed in the Cochrane Central Register of Controlled Trials (CENTRAL) are also captured in PubMed and Web of Science. Third, SPORTDiscus (exercise science), CINAHL (nursing), and PsycINFO (psychology) target niche disciplinary domains; while they may contain a small number of discipline-specific RCTs not indexed in the three core databases, these would primarily represent gray literature, conference proceedings, or studies from low-circulation regional journals. Nevertheless, this restriction carries identifiable implications for the comprehensiveness of the evidence base. Most critically, we may have missed a small number of unpublished or gray literature RCTs, as well as trials published exclusively in regional nursing or psychology journals not indexed in the three core databases. This could potentially introduce a degree of retrieval bias, particularly for outcomes more closely aligned with nursing or psychological research (e.g., mental health, social participation). However, given that all included RCTs in this review reported core clinical and public health outcomes that are standardly indexed in PubMed and Web of Science, we judge that the omission of these niche databases is unlikely to materially alter the overall pooled effect estimates or core conclusions of the review. This limitation is further elaborated in the Discussion section.

### 2.2 Eligibility criteria

Inclusion and exclusion criteria were formulated following the PICO framework to guarantee unified, operable screening standards and avoid subjective bias. All criteria were predefined and not adjusted according to retrieved literature.

#### 2.2.1 Inclusion criteria

(a) Study design: randomized controlled trial (RCT); (b) Participants: community-dwelling older adults aged 60 years and above without chronic diseases. For the purpose of this review, “without chronic diseases” was operationalized as the absence of physician-diagnosed major chronic conditions (including cardiovascular disease, type 2 diabetes, cancer, chronic respiratory disease, and neurodegenerative disease) that would substantially limit physical function or require ongoing specialized medical care. This definition was applied consistently across all included RCTs based on their original eligibility criteria. All included trials required participants to be functionally independent and residing in community (non-institutional) settings. No upper age limit was predefined in the review protocol. The highest age observed across included primary studies was 93 years; (c) Intervention: participants in the intervention group received structured physical activity intervention encompassing general physical activity, aerobic exercise, and resistance training. Eligible interventions were required to have a clearly reported and quantifiable protocol with explicitly specified weekly frequency, session duration, and total intervention period. No uniform restrictions were imposed on intervention intensity, weekly frequency, session duration, or total intervention duration, in order to capture the full spectrum of eligible randomized controlled trials and acknowledge the inherent heterogeneity of physical activity protocols in real-world settings; (d) Comparator: the control group received no form of physical activity intervention (no additional physical activity) and maintained their daily routines as usual; e Outcomes: Active Ageing and physical function. Specific indicators of Active Ageing included global Active Ageing, mental health, social participation and functional health. Indicators of physical function covered the Short Physical Performance Battery (SPPB), chair stand test, Timed Up and Go test (TUG), gait speed, handgrip strength and balance capacity.

#### 2.2.2 Exclusion criteria

a Study design: non-RCT studies; b Participants: subjects failing to meet the above inclusion criteria, or trials with severe missing baseline data that prevented valid data extraction; c Interventions: ambiguous intervention protocols with unquantifiable dosage or duration, or unclear differences between intervention and control groups; d Outcomes: studies failing to report the predefined primary outcomes, or with severe missing outcome data that could not be converted or extracted; e Other types of publications: duplicate publications (only the latest or largest-sample version was retained), conference abstracts without complete raw data, dissertations if corresponding journal articles had been published, and low-quality studies with contradictory data or incoherent logic that could not undergo risk-of-bias assessment.

The eligibility criteria outlined above were applied consistently throughout the entire study screening and selection process, and were fully consistent with the PROSPERO-registered review protocol (CRD420261465400). This broad parameterization of intervention characteristics was intentionally specified in the protocol to enable a comprehensive evidence synthesis across diverse physical activity regimens, rather than limiting the review to a single fixed intervention protocol. This approach is consistent with established methodological standards for systematic reviews of physical activity interventions, which typically encompass heterogeneous delivery models, durations, and intensities.

### 2.3 Literature screening and data extraction

#### 2.3.1 Literature screening

A dual independent screening procedure was adopted. Two trained investigators (W. X. W, L. C. Y) separately screened titles and abstracts of retrieved records according to the predefined eligibility criteria to exclude clearly ineligible papers. Full texts of preliminarily qualified articles were then retrieved and read for secondary screening to finalize included studies. Disagreements between the two reviewers were resolved via discussion; unresolved discrepancies were arbitrated by a third independent researcher.

#### 2.3.2 Data extraction

Two investigators (W. X. W, L. C. Y) independently extracted core information from included studies using a standardized data extraction form. All extracted data were cross-checked to ensure accuracy. Any disagreements were resolved through discussion or arbitration by a third researcher. Extracted information covered five categories: a Basic study information: first author, publication year, country/region, sample size (intervention group/control group); b Study design: randomization method, blinding type (single-blind, double-blind, triple-blind, or no blinding), allocation concealment, follow-up duration, loss-to-follow-up rate and reasons for dropout; c Intervention and comparator details: specific protocols for intervention and control groups; d Outcome indicators: measurement tools and raw outcome data for intervention and control groups; e Quality assessment information: quality control measures described in original papers and detailed implementation of randomization procedures.

### 2.4 Quality assessment

The internationally recognized tool for RCT methodological quality evaluation was used for dual independent risk-of-bias assessment. Assessors scored or rated each study strictly in line with tool standards, cross-checked evaluation results, and resolved disagreements via discussion or a third arbitrator. The Cochrane Risk of Bias tool (RoB 1.0) was applied to all RCTs, evaluating bias across seven core domains: bias arising from the randomization process, bias due to allocation concealment, bias of participants and personnel (performance bias), bias in outcome assessment (detection bias), incomplete outcome data, and selective outcome reporting. Each domain was rated as low risk of bias, high risk of bias, or unclear risk of bias. The overall risk of bias for each trial was determined by synthesizing ratings across all domains.

### 2.5 Data synthesis and analysis

Statistical software including RevMan 5.4 and Stata 16.0 was used for data synthesis and analysis, with software versions clearly stated. All analytical procedures were predefined and conducted in full compliance with meta-analysis reporting standards.

#### 2.5.1 Heterogeneity test

Heterogeneity testing was performed on all included studies to evaluate consistency of effect sizes and avoid biased pooled results caused by excessive between-study heterogeneity. (a) Testing methods: The Chi-squared (*Q*) test and *I*^2^ statistic were adopted. A *p*-value < 0.1 in the *Q* test indicated statistically significant heterogeneity. The *I*^2^ statistic quantified the magnitude of heterogeneity: 0–25% = negligible heterogeneity, 25–50% = mild heterogeneity, 50–75% = moderate heterogeneity, >75% = substantial heterogeneity; (b) Heterogeneity management: The selection between fixed-effects and random-effects models was primarily driven by *a priori* methodological and clinical assumptions, rather than relying solely on observed *I*^2^ statistics. The fixed-effects model was prespecified for scenarios in which included trials were expected to share a common underlying true effect (i.e., identical target population, comparable intervention constructs, and consistent outcome definitions). Given the substantial between-trial variability in intervention modalities, weekly frequency, session duration, total intervention length, and measurement instruments across the present evidence base, random-effects models were the pre-specified default for most pooled analyses, assuming that true intervention effects would follow a distribution across studies. Nevertheless, the fixed-effects model could be adopted when strong *a priori* clinical, conceptual and mechanistic homogeneity supported the assumption of a single common true effect across trials, even if moderate statistical heterogeneity (*I*^2^ ≤ 50%) was observed. This criterion was prespecified based on the consistency of core constructs, intervention mechanisms, and measurement frameworks across studies, rather than *post hoc* observed heterogeneity values, and was applied to three outcomes that met all predefined conditions, namely the Short Physical Performance Battery, chair stand test and social participation. For outcomes with mild-to-moderate statistical heterogeneity (*I*^2^ = 25–75%), subgroup analyses were performed to explore potential sources of between-study variation. For outcomes with substantial-to-extreme heterogeneity (*I*^2^ > 75%), we first double-checked data extraction accuracy and evaluated study-level methodological quality. If trials shared consistent population characteristics, core intervention construct, and identical conceptual outcome definition despite statistical heterogeneity, random-effects pooling was retained, and potential sources of heterogeneity were elaborated in the Discussion section. Narrative synthesis replaced meta-analysis only when profound clinical heterogeneity rendered quantitative pooling uninterpretable. It should be emphasized that adoption of a random-effects model addresses statistical heterogeneity but does not inherently justify quantitative pooling. Beyond statistical thresholds, we judged clinical and conceptual appropriateness for pooling based on three explicit pre-specified criteria. First, studies target the same overarching construct of Active Ageing-related health outcomes. Second, studies adopt a consistent comparator condition of no supplementary physical activity intervention with routine daily living maintained. Third, study populations are community-dwelling older adults aged 60 years and above without chronic disease. When all three criteria were satisfied, quantitative meta-synthesis was retained even under extreme *I*^2^ values. Even so, pooled effect sizes from highly heterogeneous analyses should be interpreted cautiously, with attention to their conceptual plausibility and potential clinical meaning, rather than treated as definitive point estimates.

#### 2.5.2 Effect size selection and data synthesis

Appropriate effect sizes were selected according to outcome variable types. Standardized Mean Difference (SMD) was used for continuous outcomes where measurement scales varied across trials, alongside corresponding 95% confidence intervals (95%CI). This approach standardizes effect sizes to a common metric, allowing quantitative pooling of results from studies using different instruments that measure the same underlying conceptual construct. For each outcome analyzed, included studies used instruments targeting the same underlying construct, ensuring conceptual comparability for pooled SMD calculations. This pooling approach is theoretically grounded in the World Health Organization’s unified Active Ageing framework, which defines global Active Ageing, mental health, social participation, and functional health as consistent core constructs. Within each domain, all instruments operationalize the same conceptual target, and their convergent validity within each construct is well established in prior gerontological research and meta-analyses. Full mapping of each measurement instrument to its corresponding meta-analytic outcome domain is presented in Table 1. Model selection was guided primarily by clinical, conceptual, and mechanistic homogeneity assumptions predefined in our PROSPERO registered review protocol, with observed *I*^2^ statistics serving only as a secondary reference for heterogeneity quantification rather than a deterministic decision rule. Fixed effects models were pre specified for three outcomes that met the dual criteria of strong construct homogeneity and acceptable statistical heterogeneity, the Short Physical Performance Battery, chair stand test, and social participation. For the Short Physical Performance Battery and chair stand test, both targeted identical lower extremity physical function constructs, adopted standardized performance based tests with uniform scoring protocols, recruited comparable community dwelling older adult samples, and presented negligible observed statistical heterogeneity (*I*^2^ = 0% and *I*^2^ = 11%, respectively), fully supporting the assumption of a single common true intervention effect. For social participation, the fixed effects specification was justified by three interrelated lines of *a priori* evidence. First, all included measurement instruments were operationalized based on the unified WHO Active Ageing framework of social engagement, measuring the same core construct of frequency, quality, and scope of social interaction and community participation. Second, all physical activity interventions included in this analysis exerted effects on social participation primarily through the same core mechanism, group based interactive activity that provides structured social contact, role engagement, and peer support. Third, the observed moderate statistical heterogeneity (*I*^2^ = 48%) fell below the 50% threshold commonly used to distinguish mild to moderate from substantial heterogeneity, and convergent validity of the included social participation measures has been well established in prior gerontological meta analyses. Collectively, these conceptual, mechanistic and statistical considerations supported the *a priori* assumption of a common underlying intervention effect across trials for this outcome. All other outcomes were synthesized using random effects models, given expected variability in true effect sizes across diverse intervention protocols, measurement tools, and study contexts. Forest plots were generated to visually display individual study effect sizes and pooled estimates, incorporating study weights, effect sizes, 95%CIs and corresponding *p*-values for overall effect tests.

**Table 1:** Mapping of measurement instruments to meta-analytic outcome domains.

Meta-analytic outcome domain	Full instrument name	Abbreviation	Instrument type	Core construct measured
Active Ageing domain				
Global Active Ageing	Instrumental Activities of Daily Living scale	IADL	Self-report/performance scale	Overall multidimensional active aging (functional health, social participation, mental health, independence)
	The Healthy Aging Instrument	HAI	Self-report scale	Overall active aging status across health, social, and psychological dimensions
Mental health	15-item Geriatric Depression Scale	GDS-15	Self-report scale	Depressive symptoms and psychological well-being in older adults
	Geriatric Depression Scale Short Form	GDS-SF	Self-report scale	Depressive symptomatology and mood state
	36-Item Short Form Health Survey – mental health subscale	SF-36 mental subscale	Self-report subscale	General psychological well-being and mental health status
	Mental test questionnaire	AMT	Self-report questionnaire	General mental health and emotional status
Social participation	Instrumental Activities of Daily Living scale – social participation subdimension	IADL social subdimension	Self-report subscale	Frequency of social interaction and community participation
	The Healthy Aging Instrument – social participation subdimension	HAI social subdimension	Self-report subscale	Social engagement and participation in community activities
	Lubben Social Network Scale	LSNS	Self-report scale	Size of social network and level of social support
Functional health	Activities of Daily Living scale	ADL	Self-report/performance scale	Basic daily functional independence
	Instrumental Activities of Daily Living scale	IADL	Self-report/performance scale	Instrumental daily functioning and independent living capacity
	36-Item Short Form Health Survey – physical functioning subscale	SF-36 physical function subscale	Self-report subscale	Perceived physical functional ability in daily life
Physical Function domain				
Short Physical Performance Battery	Short Physical Performance Battery	SPPB	Performance test	Comprehensive lower-extremity physical function (balance, gait strength, chair stand)
Chair stand test	Chair stand test	–	Performance test	Lower-extremity muscle strength and functional mobility
Timed Up and Go test	Timed Up and Go test	TUG	Performance test	Functional mobility and dynamic balance
Gait speed	Gait speed test	–	Performance test	Walking speed and basic locomotor function
Handgrip strength	Handgrip strength test	–	Performance test	Upper-extremity muscle strength
Balance	Balance capacity assessment	–	Performance test	Static and dynamic postural balance

#### 2.5.3 Publication bias detection

Publication bias refers to the phenomenon whereby studies with significant positive results are more likely to be published, while non-significant negative findings tend to remain unpublished. Two approaches were applied for detection: (a) Funnel plot analysis: Effect sizes (e.g., RR, WMD) were plotted on the horizontal axis, and standard errors on the vertical axis. Symmetric funnel shapes indicated minimal publication bias; asymmetric distribution suggested potential publication bias; (b) Quantitative statistical test: Egger’s linear regression test was performed. At the conventional α = 0.05 level, *p* > 0.05 indicated no statistically detectable publication bias, whereas *p* < 0.05 indicated statistically significant publication bias. Interpretation of test results should account for statistical power, which is constrained by the number of included studies. If publication bias was detected, potential causes (e.g., unpublished small-sample negative trials) and its impacts on pooled findings were elaborated in the Discussion section.

## 3 Results

### 3.1 Study selection

A total of 25 eligible studies were retrieved from the three target databases. After removing 291 duplicate records, 1,115 full-text articles were screened based on titles and abstracts. Upon full-text eligibility assessment, 852 publications were excluded. Ultimately, 25 trials met all predefined criteria and were included in the present systematic review and meta-analysis. The complete study selection process is documented in the PRISMA 2020 flow diagram, which is presented as Figure 1 in the main text. A high-resolution version of the flow diagram, together with the completed PRISMA 2020 checklist and full database search output records, are provided in the Supplementary materials.

**Figure 1:**
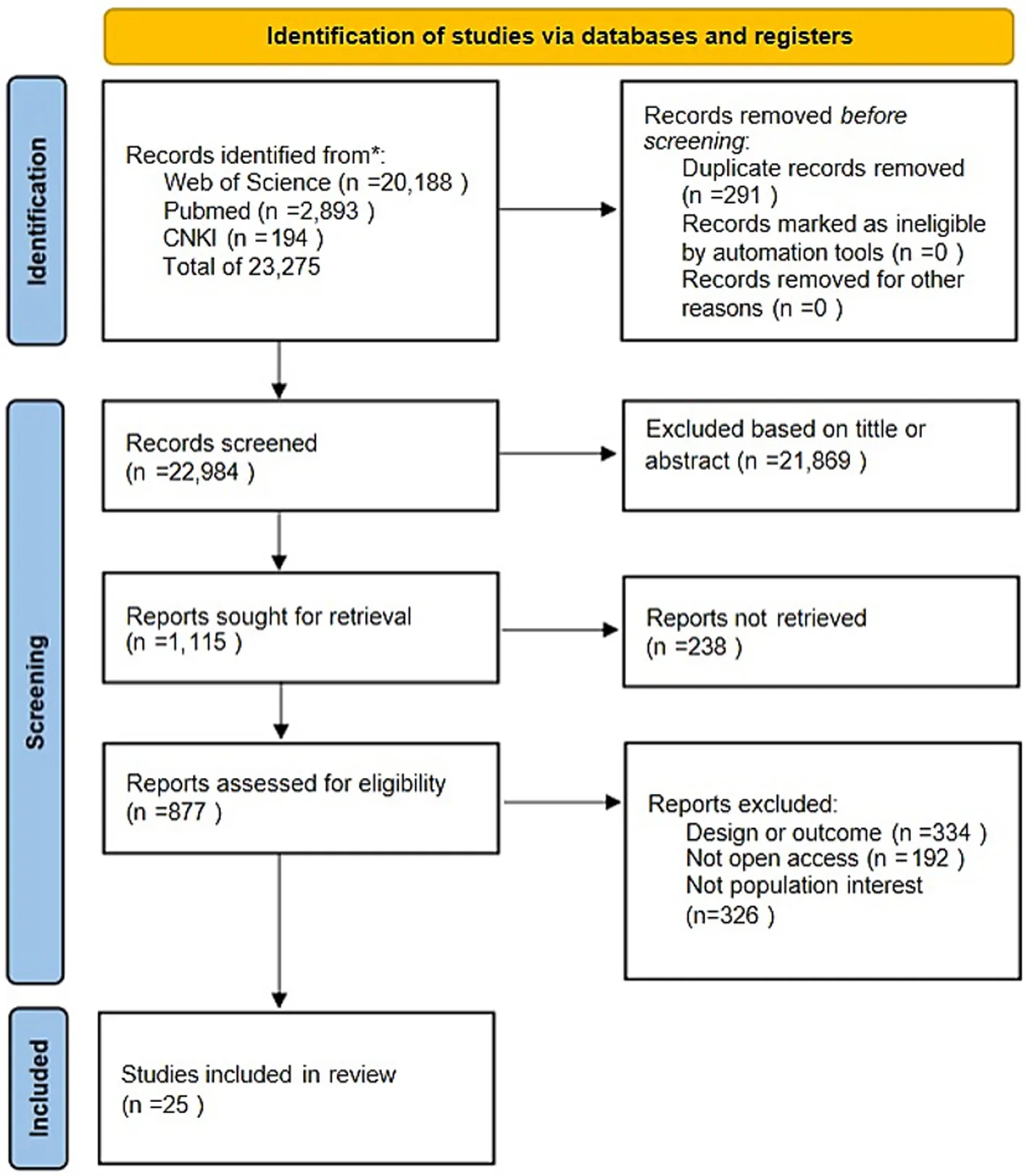
PRISMA 2020 flow diagram.

### 3.2 Characteristics of the included studies

This meta-analysis incorporated 25 randomized controlled trials (RCTs) with a pooled total sample of 7,861 participants, including 3,955 individuals in intervention groups and 3,906 in control groups. All included trials were published in English between 2005 and 2026, covering multiple regions including China, the United States, the United Kingdom and other nations. The literature search cutoff date was July 1, 2026. Participants aged 60 years and above from diverse geographical settings across Asia, Europe, North America and South America were enrolled, with the maximum reported participant age among included trials reaching 93 years, providing broad geographic coverage. Nevertheless, the sample cannot be considered fully representative of the global older adult population given the limited number of included countries and uneven distribution of study locations.

General characteristics of included trials were as follows: all participants were older adults aged 60–93 years. Intervention and control groups across all trials exhibited comparable gender composition and age distribution, demonstrating satisfactory baseline homogeneity. This minimized confounding bias originating from baseline disparities in intervention effect estimates.

Regarding intervention protocols, all intervention groups received structured Physical Activity Intervention, which fell into three modalities: general group physical activity (e.g., square dance), aerobic exercise (e.g., yoga), and resistance training. Specifically, 15 trials adopted general physical activity programs, 7 implemented aerobic exercise, and 3 applied resistance training. Intervention frequency ranged from one to seven sessions weekly, with single-session duration spanning 20 to 150 min and total intervention periods from 1 week to 12 months. This wide variation in intervention parameters was fully aligned with the predefined eligibility criteria, which did not set uniform thresholds for frequency, session duration, total period, or intensity.

Interventions were primarily delivered in community and domestic settings, with some sessions supervised by professional fitness instructors. Control group protocols fell into two main categories: no supplementary physical activity (participants only received routine classroom education), or general health education irrelevant to physical exercise. Clear, comparable control interventions were reported across all trials (see Table 2: baseline characteristics of included studies). Notably, several included trials failed to report complete data on exercise intensity, assessment instruments or intervention duration (marked N/A). Such missing data may limit the interpretability of findings from subgroup analyses and meta-regression, constituting a minor limitation of this review.

**Table 2:** Characteristics of enrolled studies.

Order number	Inclusion criteria	Area	Participant sample (n)	Sex (Male, n, %)	Age (*M* ± SD)	Intervention method	Intensity	Intervention time	Survey tools	Time of duration (week)	Outcome
1	(Beyer et al., 2015) RCT	Germany	IG:2367 CG:2367	IG: 1354(57.2%) CG:1354(57.2%)	65~ (Range)	IG: General physical activity CG: No exercise intervention	N/A	2.5 h per week	SF-36 IPAQ	48	Functional Health
2	(Chi et al., 2021) RCT	China	IG:95 CG:100	IG: 22(23.2%) CG: 36(36.0%)	65~ (Range)	IG: General physical activity CG: No exercise intervention	N/A	1 h per session, twice weekly	IPAQ	19	Functional Health Global Active Ageing Mental Health Social Participation
3	(Davodi, 2023) RCT	Iran	IG:30 CG:30	IG: 16(53.3%) CG: 13(43.3%)	IG:66.5 ± 5.1 CG:66.4 ± 4.4	IG: General physical activity CG: No exercise intervention	N/A	1 h per session, three times weekly	AMT ADL IADL	6	Social Participation Global Active Ageing
4	(Mendoza-Ruvalcaba and Arias-Merino, 2015) RCT	Mexico	IG:31 CG:33	IG:2(6.5%) CG:5(15.2%)	IG:70.45 ± 6.37 CG:70.82 ± 7.20	IG: General physical activity CG: No exercise intervention	N/A	1 h per session, twice weekly	ADL	24	Functional Health Mental Health
5	(Battaglia et al., 2016) RCT	Italy	IG:17 CG:13	IG:0(0%) CG:0(0%)	IG:68.35 ± 6.04 CG:69.69 ± 7.94	IG: General physical activity CG: No exercise intervention	N/A	1 h per session, twice weekly	Seca SF-36	8	Functional Health Mental Health
6	(Lai et al., 2022) RCT	China	IG:22 CG:14	IG:5(22.7%) CG:9(64.3%)	IG:60.05 ± 4.24 CG:59.79 ± 5.25	IG: General physical activity CG: No exercise intervention	N/A	1 h per session, three times weekly	SF-36	1	Mental Health
7	(Macdonald et al., 2024) RCT	Canada	IG:17 CG:15	IG:5(29.4%) CG:2(13.3%)	IG:70 ± 4 CG:72 ± 5	IG: General physical activity CG: No exercise intervention	N/A	1 h per session, three times weekly	Seca GDS-SF	8	Mental Health
8	(Liu and Lachman, 2021) RCT	China	IG:28 CG:28	IG:14(50%) CG:14(50%)	IG:65.4 ± 5.31 CG:65.4 ± 5.31	IG: General physical activity CG: No exercise intervention	N/A	20 min per session, seven times weekly	LSNS	4	Social Participation
9	(Yang et al., 2025) RCT	China	IG:30 CG:30	IG:10(33.3%) CG:13(43.3%)	60~ (Range)	IG: General physical activity CG: No exercise intervention	N/A	1 h per session, twice weekly	HAI	12	Functional Health Social Participation Global Active Ageing
10	(Song et al., 2026) RCT	Poland	IG:75 CG:75	N/A	IG:70.77 ± 5.62 CG:69.85 ± 5.82	IG: Square dance CG: No exercise intervention	N/A	1 h per session, three times weekly	GDS-15	12	Social Participation
11	(Antunes et al., 2005) RCT	Brazil	IG:23 CG:23	IG:23(100%) CG:23(100%)	IG:65.86 ± 3.80 CG:68.08 ± 5.49	IG: aerobic exercise CG: No exercise intervention	N/A	1 h per session, three times weekly	SF-36 GDS-15	24	Social Participation Mental Health Global Active Ageing
12	(Figueira et al., 2012) RCT	Brazil	IG:35 CG:35	IG:9(25.71%) CG:12(34.29%)	IG:68.66 ± 5.93 CG:69.80 ± 8.05	IG: General physical activity CG: No exercise intervention	Moderate	1 h per session, twice weekly	SF-36	12	Social Participation
13	(Pedersen et al., 2017) RCT	Denmark	IG:51 CG:21	IG:9(17.64%) CG:6(28.57%)	IG:79.2 ± 6.6 CG:81.3 ± 5.1	IG: resistance training CG: No exercise intervention	Moderate	1 h per session, twice weekly	SF-36 GDS-15	12	Mental Health Global Active Ageing
14	(Santanasto et al., 2017) RCT	America	IG:818 CG:817	IG:271(33%) CG:267(33%)	IG:78.7 ± 5.2 CG:79.1 ± 5.2	IG: General physical activity CG: No exercise intervention	Moderate	2.5 h per session, twice weekly	ADL	26	Balance Gait Speed Handgrip Strength
15	(Yoon et al., 2018) RCT	Korea	IG:20 CG:23	IG:6(30%) CG:7(30.4%)	IG:73.82 ± 4.37 CG:74.03 ± 4.27	IG: resistance training CG: No exercise intervention	N/A	N/A	GDS-15 IPAQ	16	SPPB TUG Gait Speed Handgrip Strength
16	(Sadjapong et al., 2020) RCT	Thailand	IG:32 CG:32	IG:16(50.0%) CG:9(28.1%)	IG:78.87 ± 1.32 CG:76.68 ± 1.14	IG: resistance training CG: No exercise intervention	Moderate to high intensity	1 h per session, three times weekly	SF-36	24	Handgrip Strength Balance TUG
17	(Roberts et al., 2017) RCT	America	IG:84 CG:69	IG: 23(27.4%) CG:14(20.3%)	IG:70.01 ± 4.98 CG:70.72 ± 4.59	IG: General physical activity CG: No exercise intervention	N/A	N/A	SF-36	24	SPPB
18	(Tew et al., 2017) RCT	UK	IG:21 CG:26	IG: 0(0%) CG:4(15.38%)	IG:73.8 ± 6.5 CG:75.7 ± 7.9	IG: Yoga CG: No exercise intervention	N/A	1.5 h per session, once weekly	EQ-5D-5L	12	SPPB Balance
19	(Gothe and McAuley, 2016) RCT	America	IG:61 CG:57	IG:12(19.7%) CG:14(24.6%)	IG:62.1 ± 5.82 CG:62.0 ± 5.39	IG: Yoga CG: No exercise intervention	Moderate	1 h per session, three times weekly	IPAQ	8	Balance Chair Stand Test Performance Gait Speed
20	(Yao and Tseng, 2019) RCT	China	IG:16 CG:15	IG:0(0%) CG:0(0%)	IG:76.38 ± 6.09 CG:78.67 ± 6.19	IG: Yoga CG: No exercise intervention	N/A	2 h per session, twice weekly	IPAQ	12	Balance Handgrip Strength
21	(Wang, 2014) RCT	UK	IG:8 CG:10	IG:0(0%) CG:2(20%)	IG: 75.5 ± 9.24 CG: 74.5 ± 8.09	IG: Yoga CG: No exercise intervention	N/A	1 h per session, twice weekly	N/A	4	Balance
22	(Noradechanunt et al., 2017) RCT	Australia	IG:13 CG:13	IG:4(30.8%) CG:3(23.1%)	IG:67.2 ± 8.3 CG:65.2 ± 6.7	IG: Yoga CG: No exercise intervention	N/A	1.5 h per session, twice weekly	SF-36 PARQ	12	Chair Stand Test Performance Gait Speed
23	(Williams et al., 2015) RCT	America	IG:7 CG:6	IG:0(0%) CG:2(20%)	IG:82.2 ± 5.2 CG:85.3 ± 4.7	IG: General physical activity CG: No exercise intervention	Moderate	N/A	N/A	8	Balance Gait Speed
24	(Tiedemann et al., 2013) RCT	Australia	IG:27 CG:27	IG:5(18%) CG:6(22%)	IG:67.7 ± 7.2 CG:67.3 ± 6.1	IG: Yoga CG: No exercise intervention	From low to high intensity	1 h per session, twice weekly	SF-36 PARQ	12	Balance Gait Speed
25	(Jorgensen et al., 2013) RCT	Denmark	IG:27 CG:30	IG:8(29.63%) CG:9(30%)	IG:75.9 ± 5.7 CG:73.7 ± 6.1	IG: General physical activity CG: No exercise intervention	High intensity	0.5 h per session, twice weekly	N/A	10	TUG Chair Stand Test Performance

Physical Activity Questionnaire (IPAQ); mental test questionnaire (AMT); activities of daily living scale (ADL); instrumental activities of daily living scale (IADL); electronic scale (SECA); Geriatric Depression Scale Short Form (GDS-SF); 18-item version of the Lubben Social Network Scale (LSNS); The Healthy Aging Instrument (HAI); 15-item Geriatric Depression Scale (GDS-15); Composite physical function or Short Physical Performance Battery (SPPB); Timed Up and Go Test (TUG); self-administered measure of health status (EQ-5D-5L); Physical Activity Readiness Questionnaire (PARQ); Short Form-36 Health Survey (SF-36); N/A = Not available; relevant data were not reported in the original studies.

All included trials reported the primary pre-specified outcomes of this meta-analysis, with outcome measurements mapped to two core conceptual domains as follows. For the Active Ageing domain, 13 studies employed the Instrumental Activities of Daily Living (IADL) scale, which covers four subdimensions of global Active Ageing, mental health, social participation and functional health. Additional measures of mental health included the Geriatric Depression Scale in both 15-item and short-form versions, the mental health subscale of the 36-Item Short Form Health Survey, and the mental test questionnaire. Social participation was also assessed using the Lubben Social Network Scale and the Healthy Aging Instrument. For the physical function domain, measures included both self-reported and performance-based instruments. Self-reported physical function was measured via the International Physical Activity Questionnaire and the physical function subscale of the 36-Item Short Form Health Survey. Objective performance-based tests included the Short Physical Performance Battery, chair stand test, Timed Up and Go test, gait speed test, handgrip strength test, and balance capacity assessment. Full details of the measurement instrument used for each outcome in each included study are provided in Table 2. All outcome measurements adopted standardized evaluation criteria with complete extractable raw data, providing robust empirical evidence to support the execution of this meta-analysis.

### 3.3 Risk of bias assessment

Figure 2 summarizes the risk-of-bias assessment results. Overall, the risk of bias across all 25 included trials was within an acceptable range. Random sequence generation was adequately implemented in 65% of the 25 trials, while adequate allocation concealment was achieved in 70% of trials. Forty-five percent of trials applied blinding to participants and research personnel, and 36% implemented blinding for outcome assessors; these trials were rated as having a low risk of detection bias. No trials (100%) presented incomplete outcome data or selective outcome reporting. Accordingly, all trials were judged to have a low risk of reporting bias.

**Figure 2:**
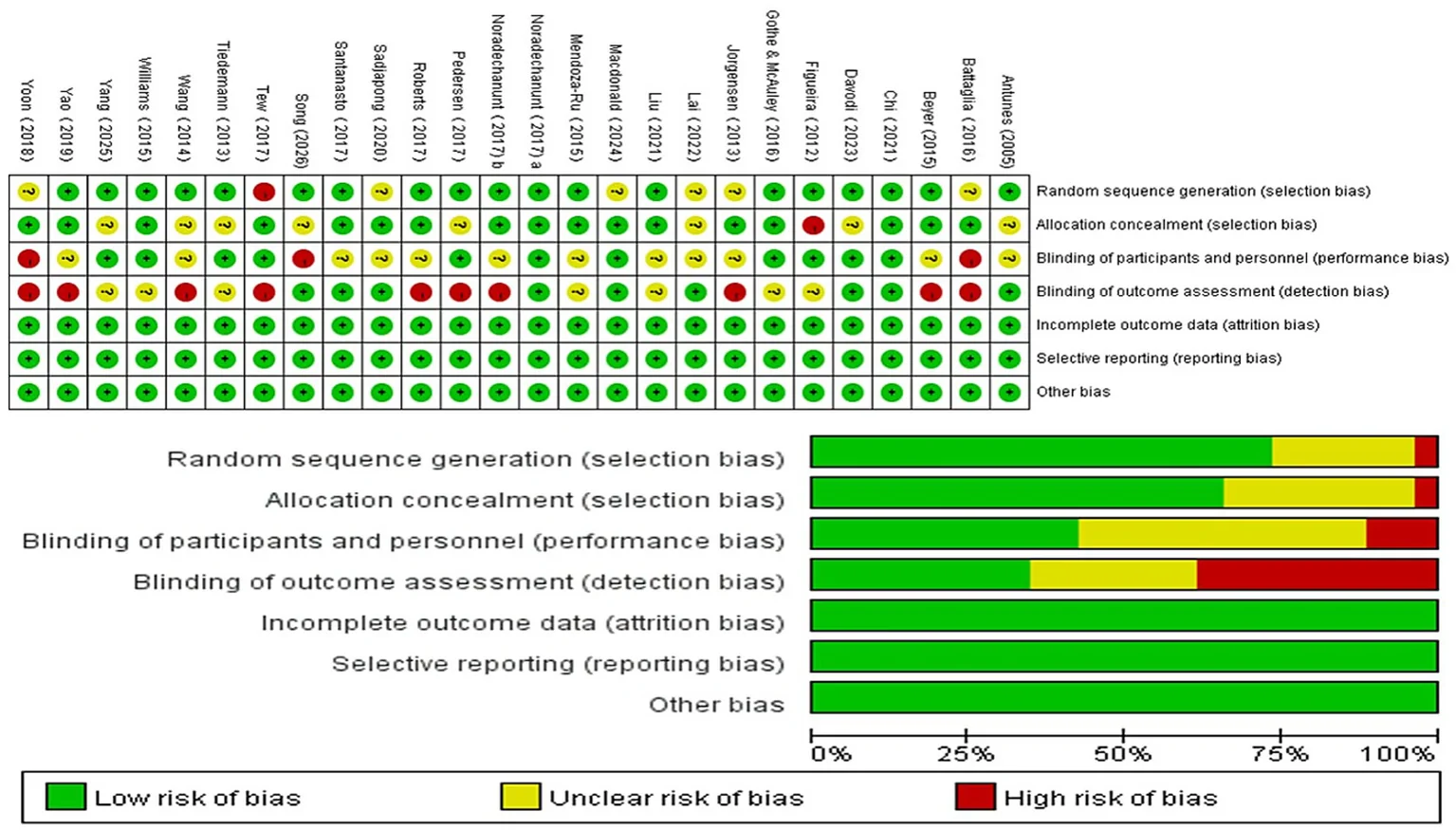
Risk of bias summary plot.

### 3.4 Results of meta-analysis

#### 3.4.1 Effects of physical activity intervention on active aging among older adults

##### 3.4.1.1 Effects of physical activity intervention on global active aging

Five studies (Antunes et al., 2005; Chi et al., 2021; Davodi, 2023; Pedersen et al., 2017; Yang et al., 2025) involving a total of 433 participants were pooled to assess scores of global Active Ageing. Heterogeneity test results indicated substantial between-study heterogeneity (*I*^2^ = 92%, *p* < 0.00001), therefore a random-effects model was adopted for pooled analysis. The pooled standardized mean difference (SMD) was 1.38 (95%CI = [0.54, 2.22], *p* = 0.001), showing that the intervention group achieved significantly higher global Active Ageing scores than the control group (*p* < 0.05). This indicated that physical activity intervention significantly improves global Active Ageing in older adults.

##### 3.4.1.2 Effects of physical activity intervention on mental health

Seven studies (Antunes et al., 2005; Battaglia et al., 2016; Chi et al., 2021; Lai et al., 2022; Macdonald et al., 2024; Mendoza-Ruvalcaba and Arias-Merino, 2015; Pedersen et al., 2017) with a combined sample of 475 participants reported mental health outcomes. Substantial heterogeneity was detected (*I*^2^ = 77%, *p* = 0.0003), hence the random-effects model was applied. The pooled SMD was 0.6 (95%CI = [0.17, 1.03], *p* = 0.006). Mental health levels were significantly higher in the intervention group than in controls (*p* < 0.05), indicating a beneficial effect of physical activity on older adults’ mental health.

##### 3.4.1.3 Effects of physical activity intervention on social participation

Seven studies (Antunes et al., 2005; Chi et al., 2021; Davodi, 2023; Figueira et al., 2012; Liu and Lachman, 2021; Song et al., 2026; Yang et al., 2025) enrolling 637 participants measured social participation. Mild between-study heterogeneity was observed (*I*^2^ = 48%, *p* = 0.07). Mild between study heterogeneity was observed (*I*^2^ = 48%, *p* = 0.07). In line with the prespecified analytical strategy grounded in conceptual, mechanistic and statistical homogeneity criteria for the social participation construct, a fixed-effects model was used for data pooling. The pooled SMD reached 1.03 (95%CI = [0.87, 1.20], *p* < 0.00001). Social participation scores were significantly elevated in the intervention group compared with the control group (*p* < 0.05), suggesting that physical activity intervention remarkably enhances social participation among older adults (Figure 3).

**Figure 3:**
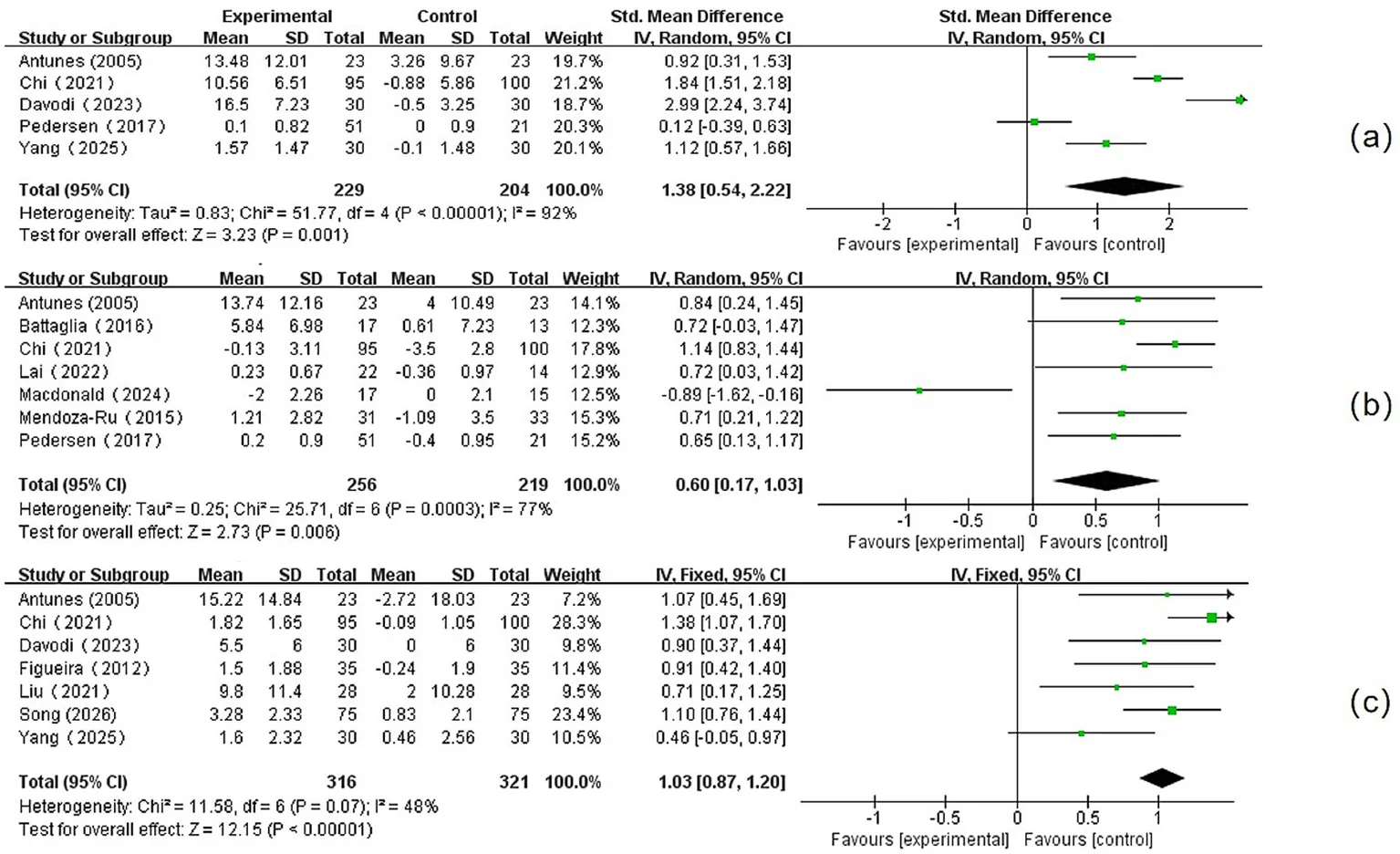
Forest plots illustrating the effects of physical activity intervention on Active Ageing in older adults: **(a)** global active aging; **(b)** mental health; **(c)** social participation.

##### 3.4.1.4 Effects of physical activity intervention on functional health

Five studies (Battaglia et al., 2016; Beyer et al., 2015; Chi et al., 2021; Mendoza-Ruvalcaba and Arias-Merino, 2015; Yang et al., 2025) including 5,083 participants reported functional health indicators. Extremely high heterogeneity was identified (*I*^2^ = 97%, *p* < 0.00001), and the random-effects model was utilized. The pooled SMD was 0.86 (95%CI = [−0.07, 1.79], *p* = 0.07). No statistically significant intergroup difference was detected (*p* > 0.05), which implied that physical activity intervention produces no significant improvement in functional health for older adults (Figure 4).

**Figure 4:**
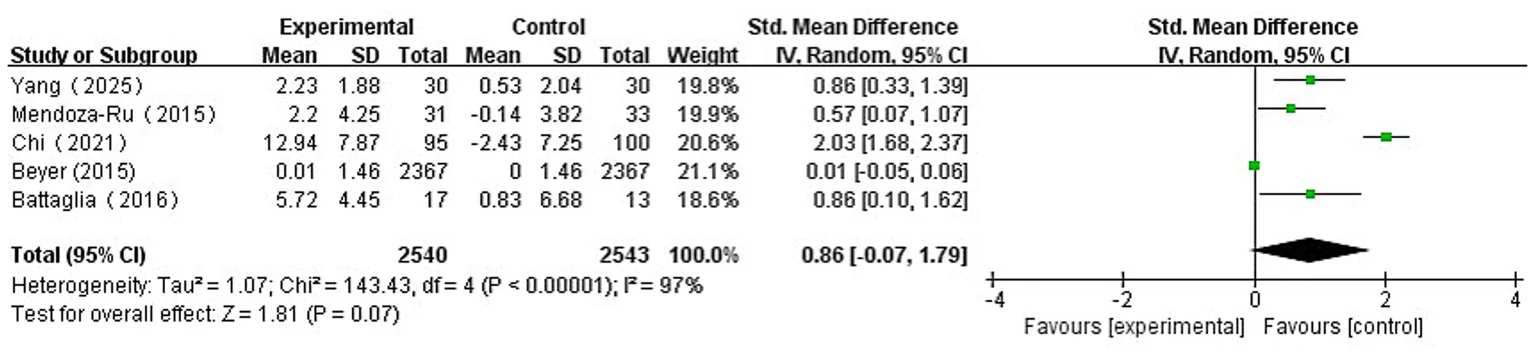
Forest plot for the effects of physical activity intervention on functional health, presenting pooled Standardized Mean Differences (SMD) and corresponding 95% Confidence Intervals (95%CI).

#### 3.4.2 Effects of physical activity intervention on physical function among older adults

##### 3.4.2.1 Effects of physical activity intervention on the short physical performance battery (SPPB)

Three studies (Roberts et al., 2017; Tew et al., 2017; Yoon et al., 2018) with a total of 243 participants reported SPPB scores. No obvious heterogeneity existed across trials (*I*^2^ = 0%, *p* = 0.91), thus the fixed-effects model was selected. The pooled SMD was 0.33 (95%CI = [0.07, 0.58], *p* = 0.01). The intervention group exhibited statistically superior SPPB performance versus controls (*p* < 0.05), indicating that physical activity intervention significantly boosts comprehensive physical function measured by SPPB in older adults.

##### 3.4.2.2 Effects of physical activity intervention on chair stand test performance

Three trials (Gothe and McAuley, 2016; Jorgensen et al., 2013; Noradechanunt et al., 2017) covering 227 participants assessed chair stand test performance. Minor heterogeneity was found (*I*^2^ = 11%, *p* = 0.34), and the fixed-effects model was adopted. The pooled SMD was 0.37 (95%CI = [0.10, 0.63], *p* = 0.006). The intervention group obtained significantly better chair stand test results than the control group (*p* < 0.05), demonstrating that physical activity intervention markedly improves chair stand performance in older adults.

##### 3.4.2.3 Effects of physical activity intervention on the timed up and go (TUG) test

Three studies (Jorgensen et al., 2013; Sadjapong et al., 2020; Yoon et al., 2018) involving 164 participants reported TUG outcomes. Moderate heterogeneity was detected (*I*^2^ = 56%, *p* = 0.10), so the random-effects model was used. The pooled SMD was −0.68 (95%CI = [−1.16, −0.20], *p* = 0.006). TUG time was statistically significantly lower in the intervention group (*p* < 0.05), which meant physical activity intervention effectively enhances mobility performance measured by TUG for older adults (Figure 5).

**Figure 5:**
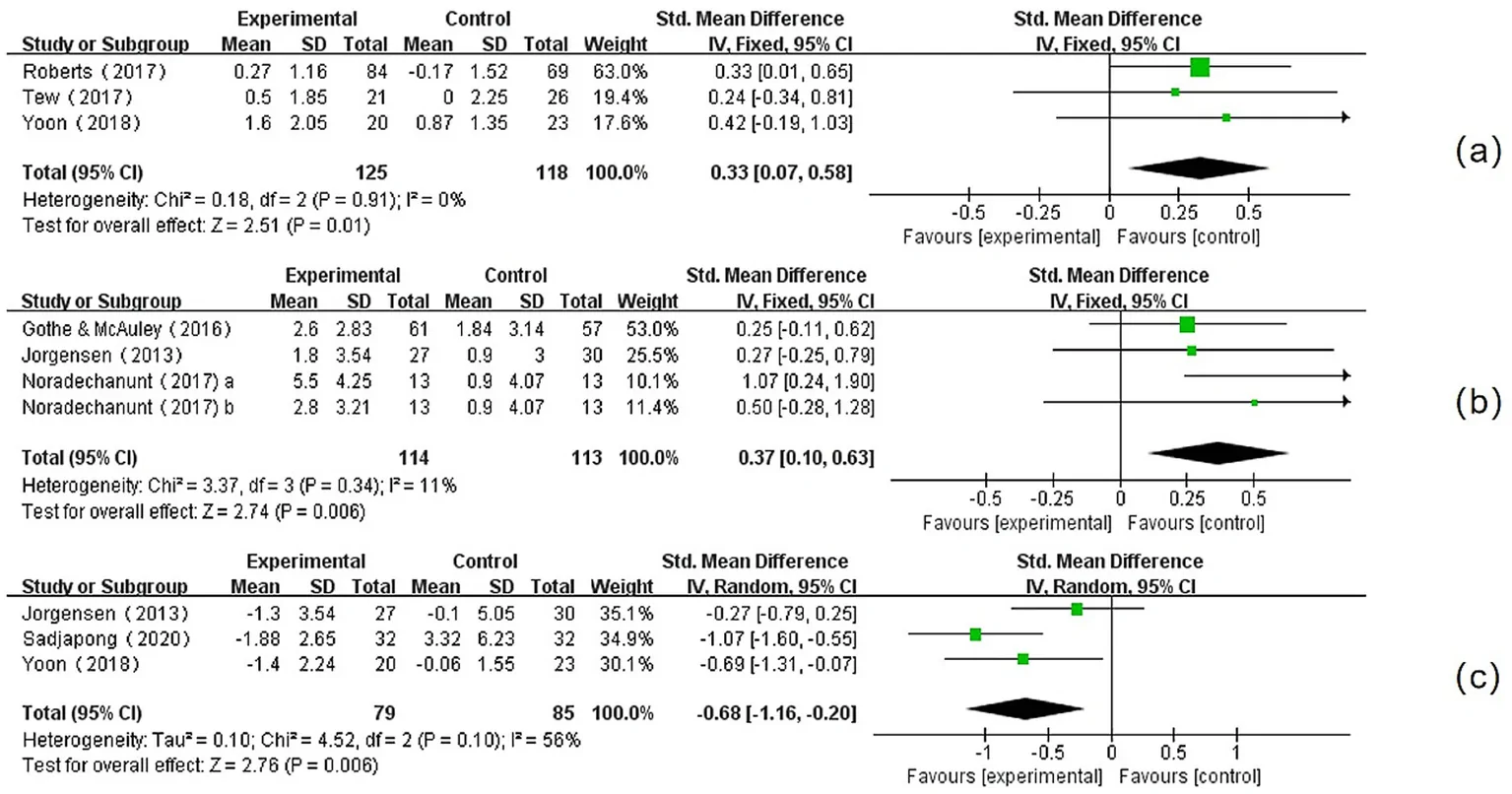
Forest plots illustrating the effects of physical activity intervention on physical function in older adults: **(a)** short physical performance battery (SPPB); **(b)** chair stand test performance; **(c)** timed up and go (TUG) test.

##### 3.4.2.4 Effects of physical activity intervention on gait speed

Six studies (Gothe and McAuley, 2016; Noradechanunt et al., 2017; Santanasto et al., 2017; Tiedemann et al., 2013; Williams et al., 2015; Yoon et al., 2018) with 1,911 participants measured gait speed. Substantial heterogeneity was present (*I*^2^ = 69%, *p* = 0.003), and the random-effects model was applied. The pooled SMD was −0.08 (95%CI = [−0.41, 0.25], *p* = 0.64). No significant between-group difference in gait speed was observed (*p* > 0.05), revealing that physical activity intervention exerts no meaningful impact on gait speed of older adults.

##### 3.4.2.5 Effects of physical activity intervention on handgrip strength

Four trials (Sadjapong et al., 2020; Santanasto et al., 2017; Yao and Tseng, 2019; Yoon et al., 2018) enrolling 1,773 participants reported handgrip strength data. Significant heterogeneity existed (*I*^2^ = 68%, *p* = 0.03), hence the random-effects model was utilized. The pooled SMD was 0.22 (95%CI = [−0.16, 0.61], *p* = 0.26). The intergroup difference in handgrip strength did not reach statistical significance (*p* > 0.05), showing that physical activity intervention fails to significantly improve handgrip strength among older adults.

##### 3.4.2.6 Effects of physical activity intervention on balance

Eight studies (Gothe and McAuley, 2016; Sadjapong et al., 2020; Santanasto et al., 2017; Tew et al., 2017; Tiedemann et al., 2013; Wang, 2014; Williams et al., 2015; Yao and Tseng, 2019) including 1,980 participants assessed balance capacity. High heterogeneity was observed (*I*^2^ = 78%, *p* < 0.0001), and the random-effects model was adopted. The pooled SMD was 0.27 (95%CI = [−0.08, 0.63], *p* = 0.13). No statistically significant difference in balance capacity was found between two groups (*p* > 0.05), indicating that physical activity intervention cannot generate significant improvements in balance for older adults (Figure 6).

**Figure 6:**
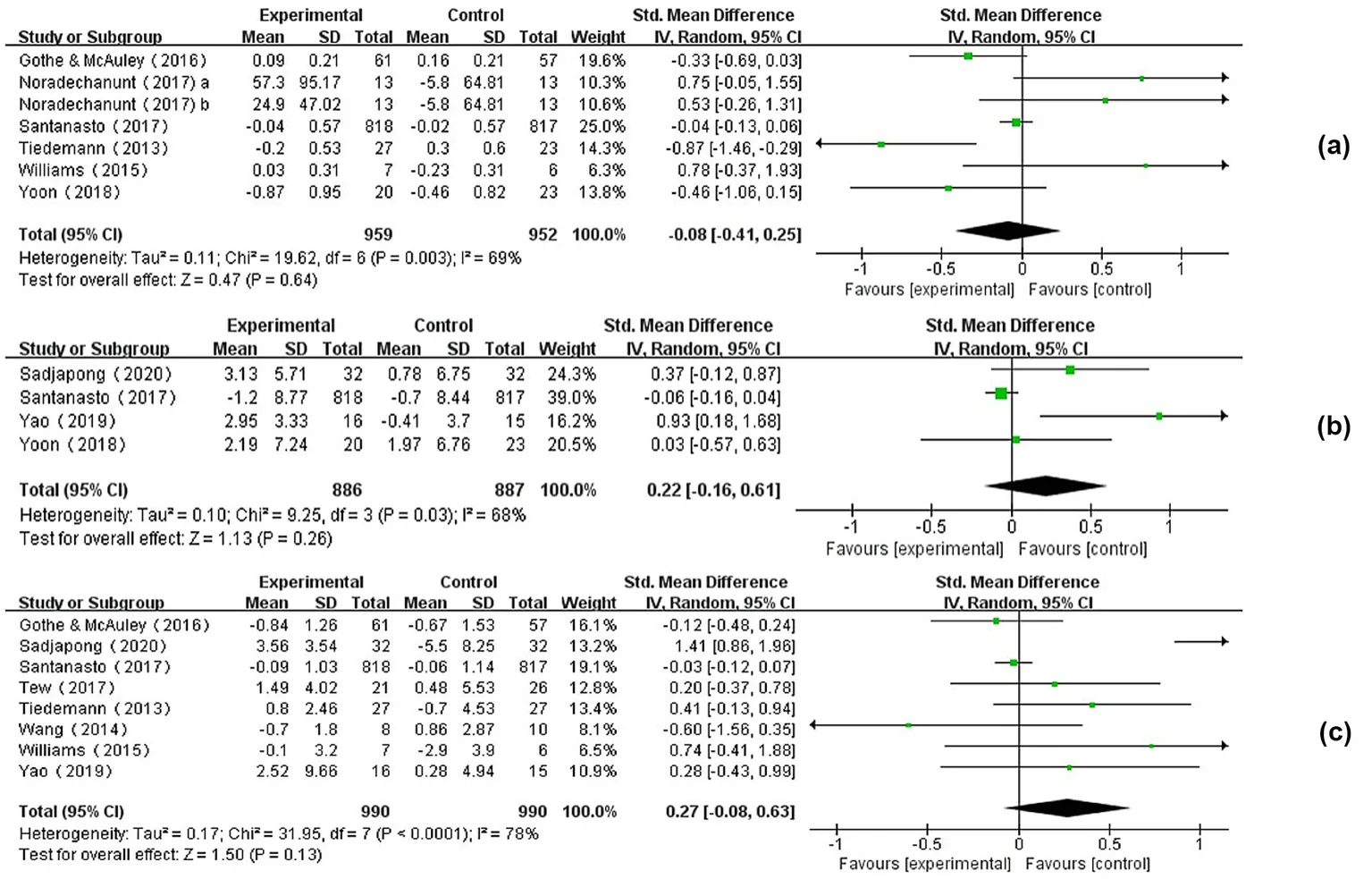
Forest plots illustrating the effects of physical activity intervention on physical function in older adults: **(a)** gait speed; **(b)** handgrip strength; **(c)** balance.

### 3.5 Subgroup analyses

The heterogeneity test indicated negligible heterogeneity for the Short Physical Performance Battery (SPPB, *I*^2^ = 0%) and chair stand test performance (*I*^2^ = 11%), and mild heterogeneity for social participation (*I*^2^ = 48%), demonstrating relatively consistent outcomes across these included trials. No statistically significant intergroup differences were detected for functional health, balance, handgrip strength and gait speed; therefore, subgroup analyses were not performed for these four outcomes. In contrast, substantial heterogeneity was observed for global Active Ageing (*I*^2^ = 92%), mental health (*I*^2^ = 77%) and the Timed Up and Go (TUG) test (*I*^2^ = 56%). Accordingly, subgroup analyses were conducted for outcomes of Active Ageing and physical function, stratified by two moderators: type of physical activity intervention and weekly intervention frequency.

#### 3.5.1 Subgroup analysis of global active aging by weekly frequency of physical activity intervention

Subgroup analyses were conducted to explore the potential moderating effect of different intervention schedules (twice weekly vs. three times weekly) on global Active Ageing among older adults. The overall meta-analysis revealed that physical activity intervention significantly improved global Active Ageing (SMD = 1.38, 95% CI [0.54, 2.22], *p* = 0.001), though extreme between-study heterogeneity was identified across all pooled effect sizes (*I*^2^ = 92%, *p* < 0.00001).

After stratification by intervention frequency, divergent intervention effects were observed between subgroups: Twice-weekly subgroup (3 effect sizes included): physical activity intervention produced a borderline statistically significant positive effect on global Active Ageing (SMD = 1.04, 95% CI [−0.01, 2.08], *p* = 0.05), with substantial within-subgroup heterogeneity (*I*^2^ = 94%, *p* < 0.0001). The 95% confidence interval crosses the null effect line, indicating that the stability of this effect is limited by the small number of included studies and insufficient statistical power in this subgroup. Three-times-weekly subgroup (2 effect sizes included): physical activity intervention also exerted marginally significant beneficial effects on global Active Ageing (SMD = 1.95, 95% CI [−0.09, 3.98], *p* = 0.06), accompanied by high within-subgroup heterogeneity (*I*^2^ = 94%, *p* < 0.0001). The test for subgroup difference indicated no statistically significant disparity in improvements of global Active Ageing between the two frequency strata (Chi^2^ = 0.61, df = 1, *p* = 0.44, *I*^2^ = 0%). Given the small number of studies within each subgroup and the non-significant between-subgroup test, these frequency-stratified results should be interpreted as exploratory descriptive findings rather than confirmatory evidence of differential effects by intervention frequency (Figure 7).

**Figure 7:**
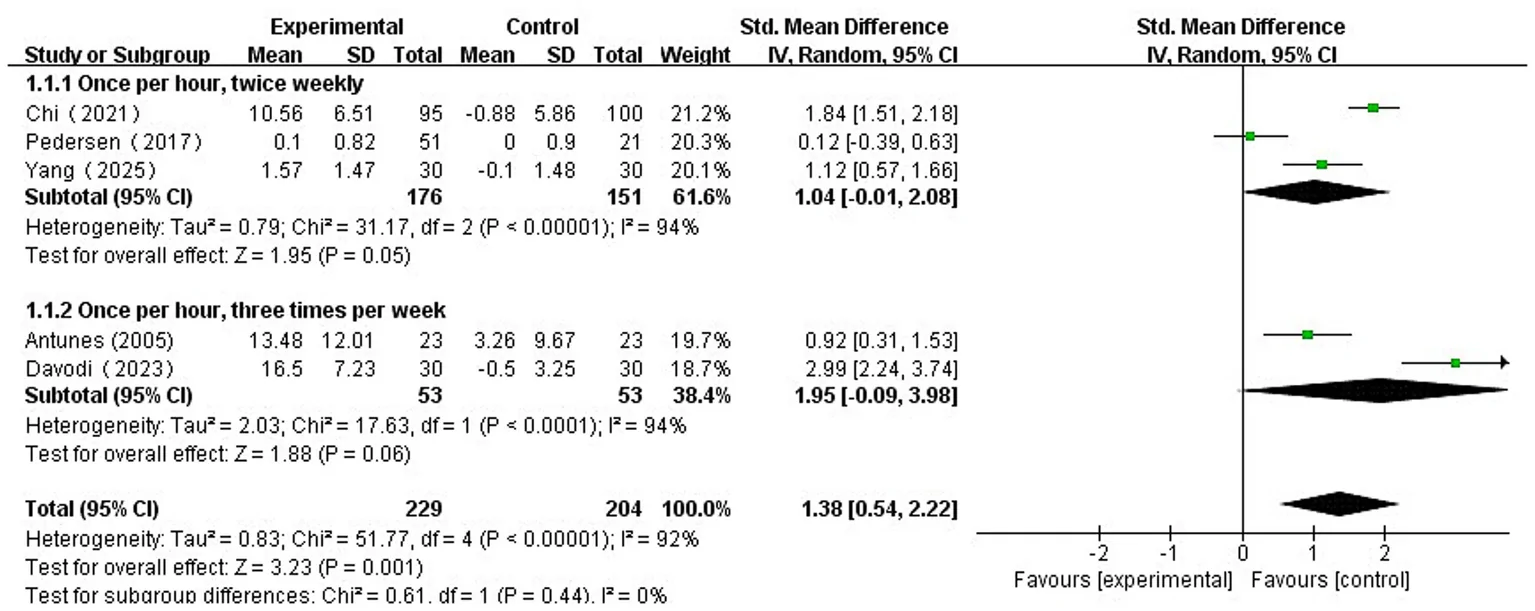
Subgroup analysis of global Active Ageing stratified by weekly intervention frequency (twice weekly vs. three times weekly). Standardized Mean Differences (SMD) and corresponding 95% Confidence Intervals (CI) are presented for each subgroup, alongside results of between-subgroup comparison.

#### 3.5.2 Subgroup analysis of mental health by weekly frequency of physical activity intervention

Subgroup analyses were performed to examine whether weekly intervention frequency (twice weekly vs. three times weekly) moderated the impacts of physical activity intervention on mental health in older adults. The overall meta-analysis demonstrated that physical activity intervention significantly improved mental health (SMD = 0.6, 95% CI [0.17, 1.03], *p* = 0.006), with substantial between-study heterogeneity across all pooled effect estimates (*I*^2^ = 77%, *p* = 0.003).

Stratified subgroup results revealed distinct intervention effects across schedules: Twice-weekly subgroup (4 effect sizes included): physical activity intervention produced statistically significant enhancements in mental health (SMD = 0.89, 95% CI [0.62, 1.16], *p* < 0.00001), with mild within-subgroup heterogeneity (*I*^2^ = 23%, *p* = 0.27). The corresponding *Q*-test *p*-value of 0.27 indicates that within-subgroup heterogeneity did not reach statistical significance. Three-times-weekly subgroup (3 effect sizes included): No statistically significant improvements in mental health were detected after intervention (SMD = 0.24, 95% CI [−0.82, 1.29], *p* = 0.66), and this subgroup presented high internal heterogeneity (*I*^2^ = 86%, *p* = 0.0007).

The test for subgroup difference did not reach statistical significance (Chi^2^ = 1.37, df = 1, *p* = 0.24, *I*^2^ = 27.0%), indicating no statistically detectable difference in the magnitude of mental health benefits between the two weekly frequency strata. In line with fundamental statistical principles, the presence of a significant within-group effect for twice-weekly interventions and a non-significant effect for three-times-weekly interventions does not by itself constitute evidence of superior efficacy for twice-weekly dosing. Given the small number of studies per subgroup and the non-significant between-subgroup test, these frequency-stratified analyses are exploratory and hypothesis-generating. The observed descriptive pattern may point to a potential moderating role of weekly intervention frequency on mental health outcomes, but this requires formal confirmation in larger, adequately powered head-to-head trials (Figure 8).

**Figure 8:**
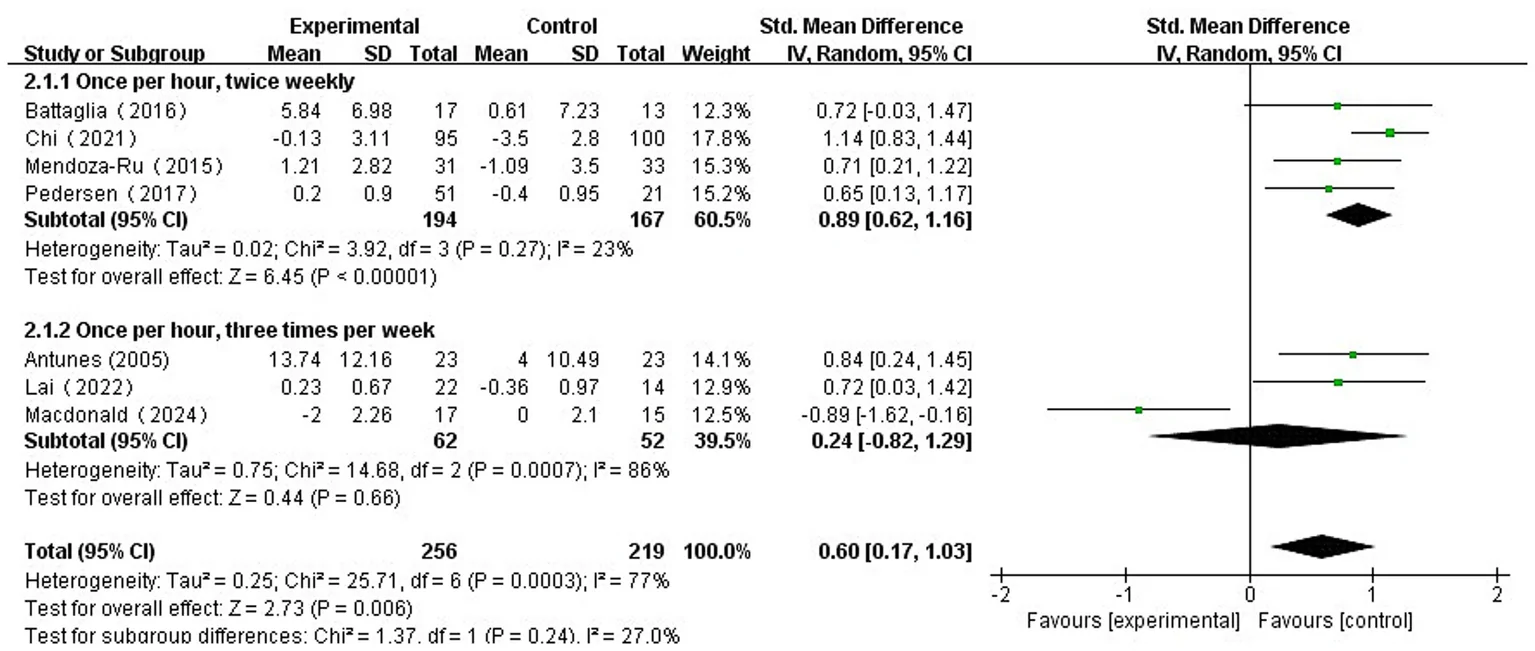
Subgroup analysis of mental health stratified by weekly intervention frequency (twice weekly vs. three times weekly). Standardized Mean Differences (SMD) and corresponding 95% Confidence Intervals (CI) are presented for each subgroup, alongside results of between-subgroup comparison.

#### 3.5.3 Subgroup analysis of social participation by type of physical activity intervention

Subgroup analyses were implemented to explore the moderating roles of distinct intervention modalities (aerobic exercise, general physical activity) on social participation among older adults, with the fixed-effects model specification retained consistently across all subgroup strata to align with the main analysis. The overall fixed-effects meta-analysis showed that physical activity intervention significantly promoted social participation (SMD = 1.03, 95% CI [0.87, 1.20], *p* < 0.00001), with mild between-study heterogeneity (*I*^2^ = 48%, *p* = 0.07).

Stratified analyses indicated varying intervention effects across activity types: Aerobic exercise subgroup (2 effect sizes included): physical activity intervention significantly boosted social participation (SMD = 1.09, 95% CI [0.79, 1.39], *p* < 0.00001), with zero within-subgroup heterogeneity (*I*^2^ = 0%, *p* = 0.93). General physical activity subgroup (5 effect sizes included): Significant improvements in social participation were also observed (SMD = 1.01, 95% CI [0.81, 1.21], *p* < 0.00001), alongside moderate-to-high within-subgroup heterogeneity (*I*^2^ = 65%, *p* = 0.02). The test for subgroup difference showed no statistically significant difference in social participation gains between aerobic exercise and general physical activity groups (Chi^2^ = 0.21, df = 1, *p* = 0.64, *I*^2^ = 0%). These findings indicate that intervention type does not appear to be a major source of heterogeneity for social participation outcomes. Given the limited number of studies in each stratum, this subgroup analysis remains descriptive and exploratory (Figure 9).

**Figure 9:**
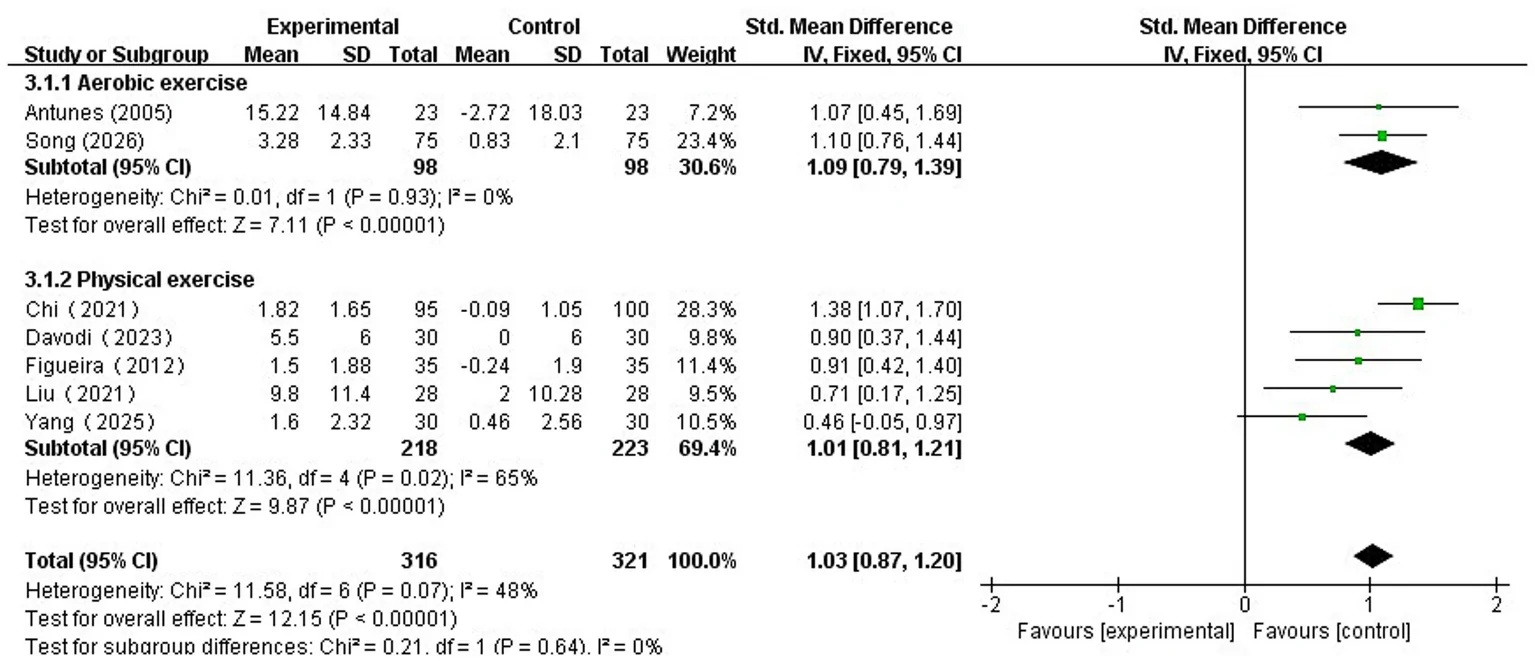
Subgroup analysis of social participation stratified by intervention type (aerobic exercise vs. general physical activity). Standardized Mean Differences (SMD) and corresponding 95% Confidence Intervals (CI) are presented for each subgroup, alongside results of between-subgroup comparison.

### 3.6 Publication bias assessment

Publication bias was assessed for all primary outcomes of physical activity intervention, including global Active Ageing, mental health, social participation, functional health, Short Physical Performance Battery (SPPB), chair stand test performance, Timed Up and Go (TUG) test, gait speed, handgrip strength and balance. Funnel plots for each outcome (Figure 10) exhibited roughly symmetrical distributions of effect sizes around the pooled estimate, with no marked visual asymmetry observed across individual plots.

**Figure 10:**
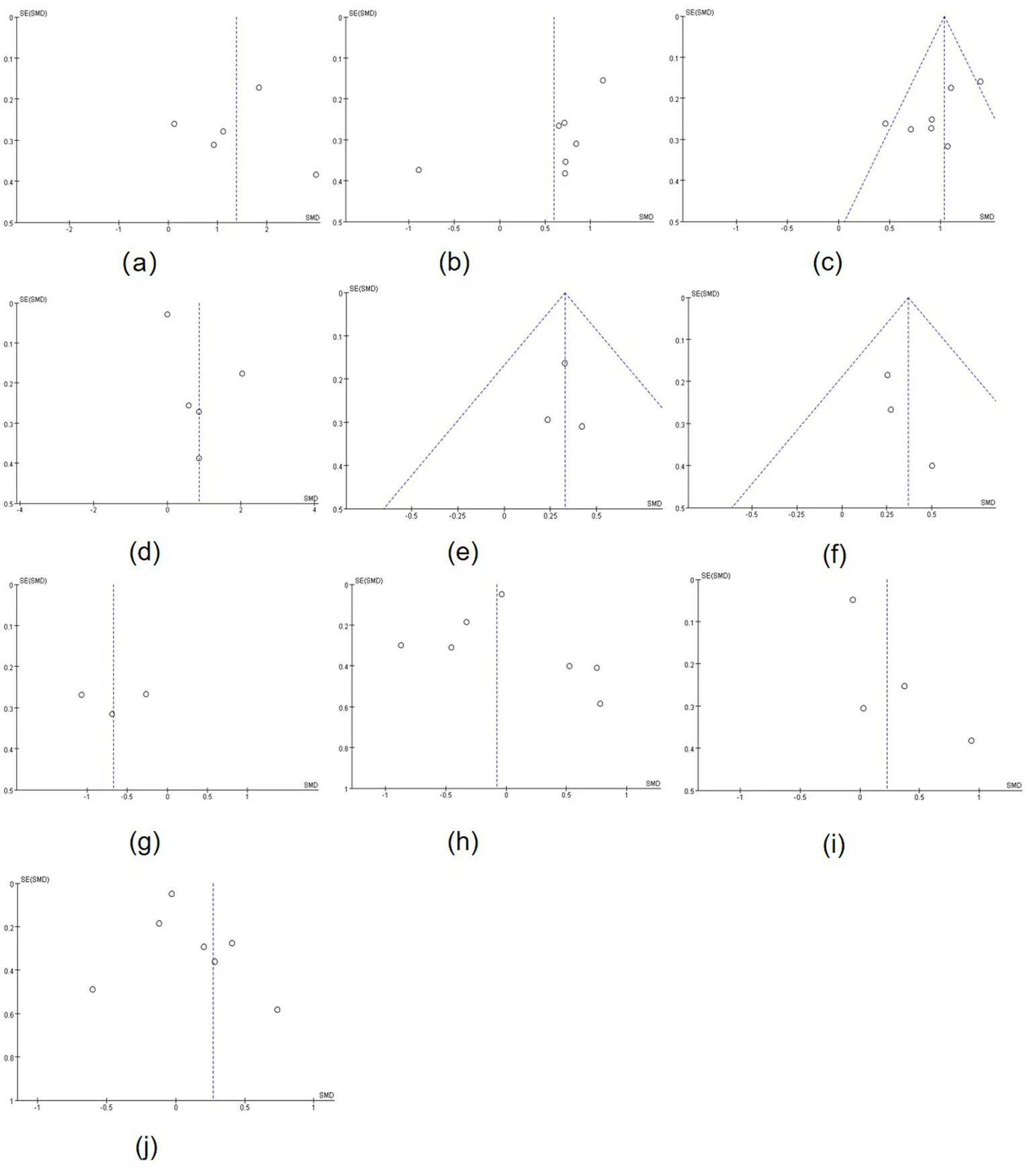
Funnel plots for detecting publication bias regarding the effects of physical activity intervention on Active Ageing and Physical function among older adults: **(a)** global active aging; **(b)** mental health; **(c)** social participation; **(d)** functional health; **(e)** short physical performance battery (SPPB); **(f)** chair stand test performance; **(g)** timed up and go (TUG) test; **(h)** gait speed; **(i)** handgrip strength; **(j)** balance.

Egger’s linear regression test was further conducted to quantify publication bias for each outcome presented in funnel plots (see Table 3). The test statistics of each indicator were listed as follows: functional health (*t* = 1.93, *p* = 0.149), global Active Ageing (*t* = −0.12, *p* = 0.913), mental health (*t* = −2.08, *p* = 0.092), social participation (*t* = −2.40, *p* = 0.062), SPPB (*t* = 0.06, *p* = 0.962), chair stand test performance (*t* = 2.10, *p* = 0.171), TUG (*t* = −0.07, *p* = 0.954), gait speed (*t* = 0.01, *p* = 0.995), handgrip strength (*t* = 2.24, *p* = 0.154), balance (*t* = 1.37, *p* = 0.221). Notably, the number of included studies per outcome ranged from 3 to 8, all below the recommended threshold of 10 studies for adequate statistical power of Egger’s regression test. For outcomes with only 3 included studies (i.e., SPPB, chair stand test, TUG), funnel plot inspection and Egger’s regression have very limited sensitivity to detect publication bias and should be interpreted as descriptive summaries rather than formal inferential tests. At the conventional *α* = 0.05 level, all outcomes yielded *p*-values greater than 0.05, indicating no statistically detectable publication bias based on Egger’s linear regression test. However, these non-significant results must not be interpreted as evidence that publication bias is absent. Formal publication bias assessments have markedly constrained interpretability given the small number of studies per analysis (ranging from 3 to 8), all below the recommended threshold of 10 studies for adequate statistical power of Egger’s test. For outcomes with only 3 included studies (i.e., SPPB, chair stand test, TUG), both funnel plot inspection and Egger’s regression have extremely limited sensitivity to detect even substantial publication bias, and these assessments should be regarded as purely descriptive with no inferential value. Across all outcomes, the possibility of residual publication bias cannot be ruled out, particularly for analyses with smaller study counts.

**Table 3:** Bias analysis.

Outcome	Egger		Begg	
	*t*	*P*	*z*	Pr
Functional Health	1.93	0.149	0.24	0.8065
Global Active Ageing	−0.12	0.913	0.24	0.8065
Mental Health	−2.08	0.092	−0.90	0.5480
Social Participation	−2.40	0.062	−1.20	0.3675
SPPB	0.06	0.962	0.00	1.0000
Chair Stand Test Performance	2.10	0.171	1.70	0.0894
TUG	−0.07	0.954	−1.04	1.0000
Gait Speed	0.01	0.995	1.50	0.1331
Handgrip Strength	2.24	0.154	1.02	0.3082
Balance	1.37	0.221	0.62	0.5362

## 4 Discussion

This systematic review systematically evaluated the impacts of physical activity intervention on four dimensions of Active Ageing and six indicators of physical function among older adults. A total of 25 randomized controlled trials (RCTs) were included. Overall analyses demonstrated that physical activity intervention yielded statistically significant improvements in global Active Ageing, social participation and mental health within the Active Ageing domain. Meanwhile, significant ameliorative effects were detected for the Short Physical Performance Battery (SPPB), chair stand test performance and Timed Up and Go (TUG) test under the physical function domain. Nevertheless, conclusive evidence regarding functional health, gait speed, handgrip strength and balance remains absent from the current body of literature.

The beneficial effects of physical activity on Active Ageing identified in this review are consistent with findings from prior meta-analyses (Chi et al., 2021; Valenzuela et al., 2023). Among all subdimensions of Active Ageing, social participation yielded the largest effect size (SMD = 1.03, *I*^2^ = 48%), with higher consistency compared with mental health and global Active Ageing. Group-based physical activities [square dance (Song et al., 2026) and group yoga (Tew et al., 2017)] inherently contain core elements of social participation, including social interaction, role identity and emotional support. Therefore, physical activity intervention generates the most prominent improvements in social participation. In contrast, mental health outcomes showed significant intervention effects accompanied by high heterogeneity (*I*^2^ = 77%). Existing evidence demonstrated that two types of interventions [aerobic exercise (Antunes et al., 2005) and general physical activity (Macdonald et al., 2024)] could alleviate depressive and anxious symptoms and optimize psychological status of Older Adults. Nevertheless, distinct disparities in intervention modes, session durations and baseline participant characteristics across these two trials led to variable effect sizes. Combined with the heterogeneity across the 7 included studies focusing on mental health outcomes in terms of intervention types [aerobic exercise (Antunes et al., 2005), general physical activity (Battaglia et al., 2016; Chi et al., 2021; Lai et al., 2022; Macdonald et al., 2024; Mendoza-Ruvalcaba and Arias-Merino, 2015), resistance training (Pedersen et al., 2017)], intervention durations (4–12 months), and mental health measurement tools [the mental subscale of SF-36 (Lai et al., 2022), depression scales, anxiety scales, etc.] such cross-study differences constitute an important source of dispersed effect sizes. In addition, despite this extreme heterogeneity, quantitative synthesis was retained because the included studies addressed the same core clinical question and shared a consistent intervention comparator framework, and the random-effects model provides a conservative pooled estimate that accounts for between study variability. The pooled result of functional health (SMD = 0.86, *p* = 0.07) was numerically close to a moderate effect size, yet the between study heterogeneity reached as high as 97%, and the confidence interval crossed the line of no effect (95% CI: −0.07 to 1.79). This extreme heterogeneity can be attributed to three key sources: first, divergent operational definitions and measurement tools of functional health across trials, including basic activities of daily living (ADL), instrumental activities of daily living (IADL), and self-reported health related function from the SF-36, which capture distinct dimensions of functional capacity; second, wide variation in baseline functional status across study populations, from fully independent community dwelling older adults to those with early functional decline; third, substantial differences in intervention modality, intensity and total duration, which produce unequal adaptive responses in functional outcomes. This suggests that existing evidence cannot support definitive conclusions regarding the effects of Physical Activity Intervention on functional health. In other words, the core question in the field of functional health is not “whether physical activity is effective”, but “under different measurement criteria and population contexts, how physical activity generates differentiated impacts on distinct types of functional health”, and current data cannot make clear judgments on this question.

Three outcomes in the present review displayed substantial-to-extreme statistical heterogeneity: global Active Ageing (*I*^2^ = 92%), functional health (*I*^2^ = 97%), and mental health (*I*^2^ = 77%). A random-effects model was applied to accommodate statistical between-study variation, yet random-effects modeling alone cannot guarantee that pooled results are clinically or conceptually appropriate. Our decision to retain quantitative synthesis for these three outcomes rested on shared conceptual and clinical features across included RCTs. All trials investigated physical-activity interventions among community-dwelling older adults without chronic diseases, employed comparable non-exercise control conditions, and measured indicators belonging to the unified Active-Ageing theoretical framework. Despite shared overarching constructs, heterogeneity originated from cross-trial differences in measurement instruments, intervention dosage, cultural framing of Active Ageing, and baseline participant status.

For global Active Ageing and mental health, the pooled SMD values pointed to moderate-magnitude beneficial effects. Nevertheless, very high heterogeneity implies that the true effect size likely varies substantially across real-world settings and study populations. The pooled SMD should therefore be regarded as an average summary estimate rather than a universal clinical effect applicable to every older-adult group. For functional health, the pooled SMD approached a moderate effect magnitude but did not reach statistical significance. Under extreme heterogeneity, this non-significant pooled finding indicates uncertainty about the true intervention effect, and we cannot draw firm inferences for clinical practice from this estimate alone. Collectively, these pooled estimates retain descriptive value for synthesizing the body of evidence, but they cannot substitute for well-powered, harmonized RCTs using unified measurement tools to produce more precise clinical effect estimates. With respect to the fixed effects specification for social participation, we acknowledge that moderate statistical heterogeneity was present across included trials. However, as outlined in our *a priori* analytical strategy, model selection was driven primarily by conceptual and mechanistic homogeneity rather than statistical heterogeneity alone. The consistent core construct of social engagement rooted in the WHO Active Ageing framework, the uniform group interaction mechanism underlying all intervention modalities, and the established convergent validity of included measures collectively supported the assumption of a common underlying intervention effect. This approach aligns with established methodological guidance for meta analyses of social gerontology outcomes, where conceptual coherence is prioritized over purely statistical heterogeneity thresholds when construct uniformity can be verified. In addition to measurement-related heterogeneity, substantial clinical heterogeneity in intervention protocols further qualifies the interpretation of pooled effect estimates. Across included studies, weekly intervention frequency ranged from 1 to 7 sessions, single-session duration from 20 to 150 min, and total intervention period from 1 week to 12 months. Furthermore, several primary studies did not fully report intervention intensity or other detailed protocol characteristics. This wide variation in intervention delivery means that pooled effect sizes represent an average estimate of the overall effect of physical activity interventions across diverse delivery models, rather than a precise effect estimate for any single standardized intervention protocol. Accordingly, caution is warranted when translating these pooled findings into specific intervention prescriptions, as different dosing regimens and implementation contexts may yield differential magnitudes of benefit.

In the dimension of physical function, the findings of this study are basically consistent with the conclusions of relevant prior studies: intervention protocols of different frequencies [1 h per session twice weekly (Wang, 2014), 1 h per session three times weekly (Sadjapong et al., 2020)] generated significant improvements in SPPB, chair stand test and TUG, while no significant improvements were observed for handgrip strength, gait speed and balance. The intervention modes adopted in this study were mainly aerobic exercise and general physical activity; only 2 included studies involved resistance training (Sadjapong et al., 2020; Yoon et al., 2018), which failed to deliver targeted stimulation for handgrip strength. In addition, for balance capacity, the primary intervention modalities among included studies were yoga (Tew et al., 2017; Tiedemann et al., 2013) and general physical activity (Williams et al., 2015). Although these interventions involved improvements in balance capacity in partial trials, balance capacity was not set as the primary training target. By contrast, improvements in balance capacity usually require structured, progressive specialized training intervention modalities. The non-significant results of gait speed in this study can be attributed to the sample composition: all included subjects were Older Adults without chronic diseases, and their baseline gait speed was already close to the normal range. Therefore, physical activity intervention can hardly generate further gains on this basis (Valenzuela et al., 2023).

Although the overall trend of this study demonstrated that physical activity intervention exerts positive promoting effects on Active Ageing and physical function among older adults, discrepancies existed in effect magnitude and the statistical significance of mental health and social participation when compared with partial prior studies. This implies that not all intervention modes and frequencies produce consistent promoting effects on Active Ageing (Chi et al., 2021). All subgroup analyses in this review should be interpreted cautiously and regarded as exploratory, given the small number of studies within most strata and the limited statistical power for formal between-subgroup comparisons. Subgroup analysis of mental health revealed a descriptive trend rather than a statistically significant between-subgroup difference. It is important to note that the presence of a statistically significant effect in one subgroup and the absence of significance in another does not, by itself, constitute evidence of a statistically significant difference between the two subgroups. Accordingly, the observation that twice-weekly interventions produced a significant within-group effect whereas three-times-weekly interventions did not cannot be used alone to conclude that twice-weekly interventions are superior. The tentative pattern of greater benefit with twice-weekly dosing may potentially be explained by accumulated fatigue, insufficient recovery, and decreased engagement associated with higher intervention frequency among older adults (Gothe and McAuley, 2016); however, this mechanistic explanation is presented as a speculative hypothesis rather than a conclusion established by the present meta-analysis, and it requires formal testing in future adequately powered head-to-head trials. In addition, subgroup analysis results for social participation showed that both aerobic exercise (Antunes et al., 2005) and general physical activity (Yang et al., 2025) generated significant beneficial effects. This indicates that the type of activity that Older Adults participate in makes no significant difference, and the core factor lies in providing opportunities for social interaction. On the contrary, heterogeneity within subgroups of global Active Ageing was not reduced after stratification (*I*^2^ = 94% for both subgroups), which demonstrates that intervention frequency is not the primary source of heterogeneity for this outcome. Potential unmeasured sources driving this extreme heterogeneity include: cross-cultural differences in the conceptualization of Active Ageing (Western samples emphasizing individual autonomy and social participation, and East Asian samples prioritizing family roles and health self-perception), inconsistent measurement instruments across studies, wide age ranges within samples (60–93 years) that may modify intervention responsiveness, and substantial variation in intervention type and total intervention duration across included trials. These factors jointly contribute to the high between-study variability, and other moderating variables need to be systematically explored in future research.

This study has the following strengths. First, this research incorporated four dimensions of Active Ageing and six indicators of physical function into a unified analytical framework. Compared with previous reviews that only focused on a single dimension, this paper can fully present the effect landscape of physical activity on the health of older adults. Second, this study only included randomized controlled trials. Relative to systematic reviews that mixed non-randomized designs, the present review achieves a higher grade of evidence and more persuasive causal inference. Third, subgroup analyses were carried out where conditions permitted, which preliminarily revealed the moderating effects of intervention frequency and intervention type on partial outcomes.

It should be noted that model selection in this meta-analysis was guided by *a priori* clinical assumptions about true-effect distribution rather than purely statistical heterogeneity thresholds. Fixed-effects models were only deployed for SPPB and chair stand test, supported by both clinical homogeneity and low observed statistical heterogeneity. Even so, residual between-trial clinical differences still existed, which may limit the generalizability of these pooled effect estimates.

Nevertheless, this study also possesses certain limitations. Firstly, substantial to extreme between-study heterogeneity was identified for multiple outcome indicators, particularly global Active Ageing (*I*^2^ = 92%), functional health (*I*^2^ = 97%) and mental health (*I*^2^ = 77%). Although a random-effects model was adopted for synthesis and subgroup analyses by intervention frequency and type were conducted for selected outcomes, the underlying sources of heterogeneity were only partially explained, and the extreme heterogeneity may reduce the stability and precision of pooled effect estimates. For social participation analyzed with a fixed-effects model, the assumption of a single common true intervention effect may carry inherent uncertainty given residual variation in study contexts, participant baseline characteristics and measurement operationalization across trials, though the preset conceptual, mechanistic and statistical criteria support the overall appropriateness of this model specification. Secondly, partial subgroups only contained a limited number of included studies in subgroup analyses, resulting in insufficient statistical power, and genuine between-subgroup differences may not be fully detected. Third, formal assessment of publication bias has substantial methodological limitations due to the small number of studies pooled for each outcome (ranging from 3 to 8). Egger’s linear regression test and visual inspection of funnel plot asymmetry have markedly reduced sensitivity when fewer than 10 studies are included. For analyses involving only three studies, formal evaluation of funnel-plot asymmetry has particularly limited value and cannot reliably rule in or rule out publication bias. While no statistically significant publication bias was detected across any outcome, these non-significant results cannot be interpreted as confirming the absence of publication bias, nor can these analyses be used to verify the stability of pooled effect estimates. The possibility of residual publication bias, particularly for outcomes with smaller study counts, cannot be ruled out. Fourth, only three core databases were searched for eligible studies, and discipline-specific databases including Scopus, Cochrane Library, SPORTDiscus, CINAHL, and PsycINFO were not included. As detailed in the Methods section, this selection was based on the high degree of journal coverage overlap between the omitted databases and the three core databases, as well as the focus of this review on core clinical and public health outcomes that are standardly indexed in PubMed and Web of Science. Nevertheless, this restriction means that we may have missed a small number of eligible RCTs published exclusively in niche discipline journals, conference proceedings, or gray literature sources not indexed in the three core databases. This could introduce mild retrieval bias, particularly for mental health and social participation outcomes that are more frequently published in psychology and social science journals. However, post-hoc reference screening of included studies and related systematic reviews did not identify additional eligible RCTs that were not captured by the core database searches, suggesting that the magnitude of this bias is likely limited. This limited database coverage may also partly account for the relatively small number of included studies for several subgroup and outcome analyses, which reduces statistical power for between-subgroup comparisons. Fifth, the eligibility criterion restricting participants to community-dwelling older adults without chronic diseases constrains the external validity and generalizability of the findings. The included sample was composed entirely of functionally independent older people free of major chronic conditions, which represents a relatively healthy subset of the broader older adult population. Accordingly, these findings cannot be directly generalized to older adults with common chronic diseases, multimorbidity, physical frailty, or those living in residential care or institutional settings. This is particularly relevant for physical function and functional health outcomes, for which baseline chronic disease and frailty status are well-established moderators of intervention response. Finally, the vast majority of included studies lacked long-term follow-up data, so this research cannot draw inferences regarding the sustained effects of physical activity intervention. Future research should conduct large-sample, multi-center randomized controlled trials with long follow-up periods, adopt unified outcome measurement tools, and fully report parameters of intervention protocols, so as to further improve evidence quality and the generalizability of research conclusions.

## 5 Conclusion

This meta-analysis demonstrates that physical activity intervention produces significant improvements in global Active Ageing, social participation and mental health within the Active Ageing domain, while its effect on functional health does not reach statistical significance. However, given the very high between-study heterogeneity observed for several outcomes, particularly global Active Ageing and functional health with *I*^2^ values exceeding 90%, the overall findings should be interpreted with caution, and the magnitude of pooled effect sizes should be regarded as indicative rather than definitive. For the physical function domain, physical activity intervention significantly enhances performance in the Short Physical Performance Battery (SPPB), chair stand test and Timed Up and Go (TUG) test, yet no significant ameliorative effects are detected for gait speed, handgrip strength and balance. Furthermore, subgroup analyses stratified by different moderating variables provided preliminary descriptive insights into potential variation in intervention effects across subgroups. However, these analyses cannot be interpreted as formal verification of the robustness of findings, as several subgroups contained only a small number of studies and had limited statistical power. Notably, subgroup analysis revealed a descriptive trend that the association between intervention frequency and mental health improvement may not follow a simple positive linear pattern. While twice-weekly interventions yielded significant within-group effects accompanied by low within-subgroup heterogeneity, three weekly sessions generated non-significant within-group outcomes. Importantly, this pattern of divergent within-group significance does not by itself demonstrate a statistically significant difference between the two frequency subgroups, so it cannot be concluded that twice-weekly interventions are superior to three-weekly interventions. This descriptive pattern indicates that higher intervention frequency may not necessarily translate into greater mental health benefits; however, since the between-subgroup difference did not reach statistical significance, weekly frequency cannot be concluded to be a significant moderator of mental health effects, and all frequency-related findings remain exploratory and hypothesis-generating. Formal publication bias tests did not detect statistically significant asymmetry across any outcome indicator via Egger’s linear regression test. However, given the small number of included studies per analysis and the resulting limited statistical power, these tests have constrained interpretability, and no definitive claim regarding the absence of publication bias can be made based on the current analyses. This study fills the research gap of meta-analyses based solely on randomized controlled trials (RCTs) in this field. It quantitatively synthesizes multi-dimensional effects of Active Ageing and physical function within a unified analytical framework and addresses the core scholarly debate regarding whether the benefits of physical activity for Active Ageing are universal or domain-specific. Nevertheless, several important limitations should be acknowledged when interpreting these findings. First, substantial to extreme between-study heterogeneity was observed for multiple outcomes, particularly global Active Ageing and functional health, which reduces the precision and stability of pooled estimates. Second, several analyses and subgroups included only a small number of studies, resulting in limited statistical power and increasing the likelihood of type II error. Third, uncertainty remains surrounding publication bias, as formal tests had constrained interpretability due to the small study counts. Fourth, included studies varied widely in intervention characteristics and outcome measurement instruments, which may contribute to the observed heterogeneity and limit direct comparison across trials. Finally, evidence regarding the long-term sustained effects of physical activity interventions remains very limited. Overall, the findings should be interpreted with appropriate caution given these methodological constraints. In terms of practical implications, the current evidence broadly supports the potential for structured physical activity interventions to enhance multiple dimensions of Active Ageing and selected physical function indicators among community-dwelling older adults without chronic diseases. However, given substantial methodological and clinical limitations — including high between-study heterogeneity, underpowered subgroup analyses, and uncertain publication bias — no definitive recommendations regarding optimal intervention modality, frequency, intensity, or duration can be made. Program design should be considered tentative and tailored to local contexts, population characteristics, and feasible intervention parameters, as confirmatory evidence from larger, well-standardized randomized controlled trials remains required. Future research should implement high-quality randomized controlled trials with large sample sizes, extended follow-up periods and fully reported intervention parameters to further verify and expand upon the current findings.

## Data Availability

The original contributions presented in the study are included in the article/Supplementary materials, further inquiries can be directed to the corresponding author.
